# Regulation of primary cilia disassembly through HUWE1-mediated TTBK2 degradation plays a crucial role in cerebellar development and medulloblastoma growth

**DOI:** 10.1038/s41418-024-01325-2

**Published:** 2024-06-15

**Authors:** I-Hsuan Lin, Yue-Ru Li, Chia-Hsiang Chang, Yu-Wen Cheng, Yu-Ting Wang, Yu-Shuen Tsai, Pei-Yi Lin, Chien-Han Kao, Ting-Yu Su, Chih-Sin Hsu, Chien-Yi Tung, Pang-Hung Hsu, Olivier Ayrault, Bon-chu Chung, Jin-Wu Tsai, Won-Jing Wang

**Affiliations:** 1 Taiwan International Graduate Program in Molecular Medicine, National Yang Ming Chiao Tung University and Academia Sinica, Taipei, Taiwan; 2 Institute of Biochemistry and Molecular Biology, National Yang Ming Chiao Tung University, Taipei, 112 Taiwan; 3 Institute of Brain Science, School of Medicine, National Yang Ming Chiao Tung University, Taipei, 112 Taiwan; 4 Institute of Molecular Biology, Academia Sinica, Taipei, 115 Taiwan; 5 Department of Life Sciences, National Central University, Taoyuan, 300 Taiwan; 6 Cancer and Immunology Research Center, National Yang Ming Chiao Tung University, Taipei, 112 Taiwan; 7 Department of Bioscience and Biotechnology, National Taiwan Ocean University, Keelung, 202 Taiwan; 8 Institut Curie, PSL Research University, CNRS UMR, INSERM, Orsay, France; 9 Université Paris Sud, Université Paris-Saclay, CNRS UMR, INSERM U, Orsay, France; 10 Graduate Institute of Biomedical Sciences, Neuroscience and Brain Disease Center, China Medical University, Taichung 404 Taiwan; 11 Advanced Therapeutics Research Center, National Yang Ming Chiao Tung University, Taipei, 112 Taiwan; 12 Brain Research Center, National Yang Ming Chiao Tung University, Taipei, 112 Taiwan

**Keywords:** CNS cancer, Cell biology, Molecular biology

## Abstract

Development of the cerebellum requires precise regulation of granule neuron progenitor (GNP) proliferation. Although it is known that primary cilia are necessary to support GNP proliferation, the exact molecular mechanism governing primary cilia dynamics within GNPs remains elusive. Here, we establish the pivotal roles for the centrosomal kinase TTBK2 (Tau tubulin kinase-2) and the E3 ubiquitin ligase HUWE1 in GNP proliferation. We show that TTBK2 is highly expressed in proliferating GNPs under Sonic Hedgehog (SHH) signaling, coinciding with active GNP proliferation and the presence of primary cilia. TTBK2 stabilizes primary cilia by inhibiting their disassembly, thereby promoting GNP proliferation in response to SHH. Mechanistically, we identify HUWE1 as a novel centrosomal E3 ligase that facilitates primary cilia disassembly by targeting TTBK2 degradation. Disassembly of primary cilia serves as a trigger for GNP differentiation, allowing their migration from the external granule layer (EGL) of the cerebellum to the internal granule layer (IGL) for subsequent maturation. Moreover, we have established a link between TTBK2 and SHH-type medulloblastoma (SHH-MB), a tumor characterized by uncontrolled GNP proliferation. TTBK2 depletion inhibits SHH-MB proliferation, indicating that TTBK2 may be a potential therapeutic target for this cancer type. In summary, our findings reveal the mechanism governing cerebellar development and highlight a potential anti-cancer strategy for SHH-MB.

## Introduction

The cerebellum, comprising the molecular layer, Purkinje cell layer, and granule cell layer, plays a pivotal role in motor coordination, memory, and cognitive processes [1, 2]. Granule neurons constitute the majority of cerebellar cells, and their development follows a well-defined sequence involving neurogenesis, differentiation, and migration. During embryonic development, granule neuron progenitors (GNPs) originate from the rhombic lip of the fourth ventricle and migrate along the cerebellar surface to form the external granule layer (EGL). During the postnatal stage, GNPs undergo an extensive proliferation phase within the EGL before exiting the cell cycle and migrating from the EGL to the internal granule layer (IGL) for further neuronal differentiation [1, 3–6].

GNP proliferation is a highly complex process. One central player in this complex process is the basic/helix-loop-helix transcription factor ATOH1. ATOH1 orchestrates the proliferative program of GNPs by regulating the transcription of downstream target genes [7, 8]. Recent research, including our own, has unveiled the role of Atoh1 in promoting GNP proliferation through primary cilia, specialized sensory organelles that transduce Sonic hedgehog (SHH) signals [9–11]. SHH ligand in developing cerebellum is secreted by Purkinje cells where it serves as a key stimulant for GNP proliferation [3, 4]. Primary cilia are predominantly observed on actively proliferating GNPs, but they are absent from differentiated granule neurons [9, 11]. Disruption of primary cilia in GNPs has been shown to impede GNP expansion, highlighting their indispensable role in cerebellar development. Another key contributor to GNP proliferation is the HECT, UBA, and WWE domain containing E3 ubiquitin ligase known as HUWE1 [12, 13]. HUWE1 has been shown to facilitate ATOH1 degradation during GNP differentiation [7, 8, 14–16]. Interestingly, mass spectrometry analyses have shown that HUWE1 is a candidate protein localizing at centrosomes [17–22], suggesting that HUWE1 may regulate centrosome-related cellular functions.

Primary cilia formation requires the recruitment of Tau tubulin kinase 2 (TTBK2) to the centrioles and its subsequent kinase activity [23–26]. Following completion of primary cilia assembly, TTBK2 is retained at centrioles, but it is released upon cilia disassembly, suggesting that presence of TTBK2 at centrioles stabilizes primary cilia. In situ hybridization studies have revealed strong expression of *TTBK2* mRNA in the cerebellum [27, 28]. Furthermore, truncation mutations in the *TTBK2* gene are associated with spinocerebellar ataxia type 11 [27, 29]. Notably, conditional *Ttbk2* knockout in adult mice results in degenerative cerebellar phenotypes, highlighting an important role of TTBK2 in cerebellum [28].

Disruption of cerebellar development has been linked to the formation of medulloblastoma (MB). MB is classified into four molecular subgroups: wingless (WNT), SHH, Group 3, and Group 4 [30–33]. Among these, SHH-type MB (SHH-MB), arising from GNPs and characterized by a constitutively active SHH signaling pathway, accounts for approximately 30% of all MB cases [30–34]. Therefore, understanding the regulatory mechanisms governing GNP proliferation is crucial for preventing this specific cancer and devising effective treatments.

In this study, we show that Ttbk2 is required for cilia maintenance and GNP proliferation during cerebellar development. We have also uncovered a novel role for HUWE1 as an E3 ligase that targets TTBK2 degradation, leading to primary cilia disassembly and the promotion of GNP differentiation. Moreover, our results reveal the importance of SHH signaling in preventing TTBK2 degradation by HUWE1, thereby maintaining primary cilia on the GNP surface to support GNP proliferation. More importantly, we demonstrate that depletion of TTBK2 impairs SHH-MB cell proliferation, representing a potential therapeutic target for this type of tumor.

## Results

### Ttbk2 is expressed at high levels in proliferating GNPs under SHH signaling

**Fig. 1:**
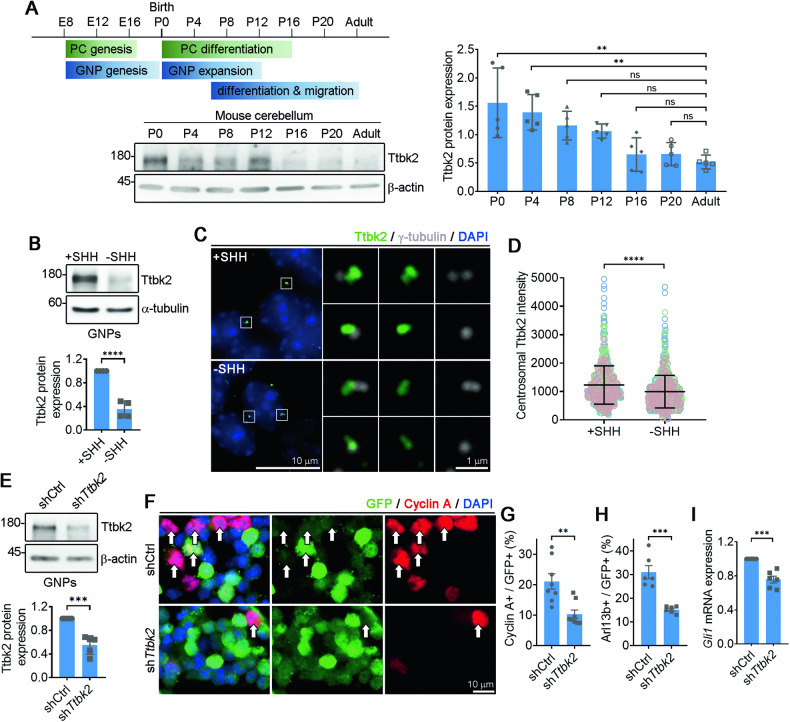
Ttbk2 expression maintains primary cilia in GNPs and promotes SHH-dependent GNP proliferation. **A** Tissue lysates from cerebellum of postnatal mouse pups and adult mice were collected and immunoblotted using antibodies against Ttbk2 and β-actin. Ttbk2 levels were quantified by normalizing with β-actin. Data were collected from n = 5 independent experiments. Error bars represent the mean ± SD. ns, not significant, **p < 0.01 by one-way ANOVA with post-hoc test. **B** Purified P7 GNPs were cultured in the presence or absence of SHH for 3 days. Immunoblots were performed to examine Ttbk2 levels with α-tubulin serving as the loading control. The Ttbk2 levels were quantified by normalizing with α-tubulin. Data were collected from n = 4 independent experiments. Error bars represent the mean ± SD. ****p < 0.0001 by Student’s t test. **C** Purified P7 GNPs in the presence and absence of SHH for 3 days were stained with anti-Ttbk2 and anti-γ-tubulin antibodies. Nuclei were stained by DAPI (blue). Regions within the marked boxes were magnified and shown in the right. Scale bars are as indicated. **D** Ttbk2 intensity was quantified. The scatter plot graph showed the results from three experiments. Each color represents an independent experiment. More than 200 cells were counted per experiment. Error bars represent the mean ± SD. ****p < 0.0001 by nonparametric test. **E** Purified P7 GNPs were infected with lentivirus carrying shCtrl or sh*Ttbk2* along with GFP for 3 days. *Ttbk2* levels were examined by immunoblots with β-actin as the loading control. Data were collected from n = 5 independent experiments. Error bars represent the mean ± SD. ***p < 0.001 by Student’s t test. **F** Immunostaining was performed to label the proliferating GNPs (Cyclin A+, red) and infected cells (GFP+). Nuclei were stained by DAPI (blue). Arrows indicate cyclin A+/GFP+ cells. Scale bar, 10 μm. **G** The percentage of cyclin A+/GFP+ cells was quantified. 200 cells from n = 8 independent experiments were tested. Error bars represent the mean ± SEM. **p < 0.01 by Student’s t test. **H** The percentage of ciliated cells in each group from Fig. S1C was quantified. 200 cells from n = 6 independent experiments were tested. Error bars represent the mean ± SEM. ***p < 0.001 by Student’s t test. **I** Purified P7 GNPs were infected with lentivirus carrying shCtrl or sh*Ttbk2* along with GFP for 3 days. The expression of *Gli1* was analyzed by qPCR. Data were normalized to an internal control (18S) and plotted as fold change above shCtrl arbitrarily set as 1. Error bars represent the mean ± SEM from n = 6 independent experiments. ***p < 0.001 by Student’s t test.

We examined Ttbk2 expression during mouse cerebellar development and observed high levels of Ttbk2 from postnatal days 0 to 4 (P0–4), followed by a gradual decrease over time (Fig. 1A). To explore this outcome further, we purified and cultured mouse GNPs either in the presence of SHH to maintain their progenitor state, or in its absence to promote GNP differentiation [35]. SHH-treated GNPs exhibited active proliferation, as confirmed by cyclin A staining (Fig. S1A). Concurrently, we observed primary cilia in 40% of SHH-treated GNPs compared to only 10% in SHH-untreated GNPs (Fig. S1B), in alignment with previous studies [9–11]. Remarkably, immunoblots showed that Ttbk2 levels were more than three-fold higher in SHH-treated GNPs compared to SHH-untreated GNPs (Fig. 1B). Moreover, we detected brighter centrosomal Ttbk2 signal in proliferating GNPs under SHH treatment, with this signal being less distinct for GNPs cultured under SHH-free conditions (Fig. 1C, D). This indicates that centrosomal Ttbk2 is maintained at high levels in proliferating GNPs under SHH signaling. The strong expression of Ttbk2 at centrosomes in proliferating GNPs also suggests that Ttbk2 may function in regulating SHH-dependent GNP proliferation through primary cilia, given TTBK2’s critical role as a kinase in ciliogenesis and the requirement for GNPs to have primary cilia for their proliferation.

### Ttbk2 maintains primary cilia and promotes SHH-dependent GNP proliferation

To ascertain Ttbk2’s involvement in GNP proliferation, we knocked down Ttbk2 in SHH-treated GNPs by transducing lentiviruses encoding short-hairpin RNA that targets *Ttbk2* (sh*Ttbk2*) or non-targeting shRNA (shCtrl) along with green fluorescent protein (GFP) (Fig. 1E). The percentage of cyclin A-positive GNPs significantly reduced in cells infected with sh*Ttbk2* compared to those with shCtrl, indicating that Ttbk2 expression supports GNP proliferation (Fig. 1F, G). Additionally, Ttbk2 knockdown significantly reduced the ciliated frequency (Figs. 1H and S1C). We also assessed the expression of the SHH downstream target gene *Gli1* using quantitative polymerase chain reaction (qPCR) and observed that Ttbk2 knockdown reduced *Gli1* expression (Fig. 1I). These findings collectively support the role of Ttbk2 in enabling GNPs to respond to the SHH, thereby facilitating GNP proliferation.

**Fig. 2:**
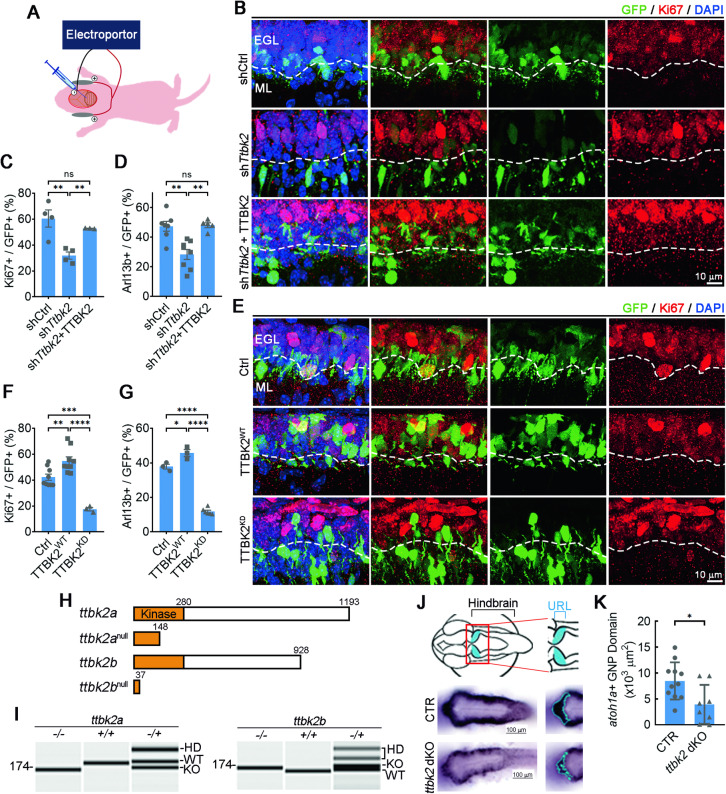
Ttbk2 controls GNP pool expansion. **A** The electroporation-based strategy for the delivery of plasmids into the EGL of P6 mice. **B** Either shCtrl, sh*Ttbk2*, or human TTBK2 expression construct along with sh*Ttbk2* was electroporated into the EGL of P6 mice followed by a waiting period of 2 days. Dashed white line distinguishes EGL (upper) and ML (lower). Immunostaining was performed to label the proliferating GNPs (Ki67+, red) and electroporated cells (GFP+). Nuclei were stained by DAPI. Scale bar, 10 μm. **C** The percentage of Ki67+/GFP+ cells in EGL was quantified. 50–200 electroporated cells were counted (n = 4 for shCtrl and sh*Ttbk2*; n = 3 for sh*Ttbk2* + TTBK2). Error bars represent the mean ± SEM. ns, not significant, **p < 0.01 by Student’s t test. **D** The percentage of Arl13b+/GFP+ cells in EGL from Fig. S2A was quantified. 50–200 electroporated cells were counted (n = 7 for shCtrl; n = 8 for sh*Ttbk2*; n = 5 for sh*Ttbk2* + TTBK2). Error bars represent the mean ± SEM. ns, not significant, **p < 0.01 by Student’s t test. **E** TTBK2^WT^, TTBK2^KD^, or control (Ctrl) was electroporated into the EGL of P6 mice followed by a waiting period of 2 days. Dashed white line distinguishes EGL (upper) and ML (lower). Immunostaining was performed to label the proliferating GNPs (Ki67+, red) and electroporated cells (GFP+). Nuclei were stained by DAPI. Scale bar: 10 μm. **F** The percentage of Ki67+/GFP+ cells in EGL was quantified. 50–200 electroporated cells were counted (n = 9 for Ctrl and TTBK2^WT^; n = 3 for TTBK2^KD^). Error bars represent the mean ± SEM. **p < 0.01, ***p < 0.001, ****p < 0.0001 by Student’s t test. **G** The percentage of Arl13b + /GFP+ cells in EGL from Fig. S2B was quantified. 50–200 electroporated cells were counted (n = 3 for Ctrl and TTBK2^WT^; n = 6 for TTBK2^KD^). Error bars represent the mean ± SEM. *p < 0.05, ****p < 0.0001 by Student’s t test. **H** Schematic diagram shows wild-type, *ttbk2a*, and *ttbk2b*, and their truncated mutants. The kinase domain is colored in yellow. **I** Genotyping of wild-type (+/+), heterozygous (−/+), or homozygous (KO; −/−) ttbk2a and ttbk2b mutant fish by capillary electrophoresis. HD: Heteroduplex band of the + and – alleles. **J** The top diagram depicts the location of zebrafish hindbrain and its precursor called URL at 2 dpf. The lower pictures are the in situ hybridization results of *atoh1a* showing the granule cell progenitor in the CTR and *ttbk2* dKO fish brain. The left picture is the whole brain, and the right picture is the cerebellar regions used for quantitation analysis. **K** Quantification of the granule progenitor domains as the area of *atoh1a*-positive domains was performed in URL of CTR and *ttbk2* dKO. n = 11 for CTR; n = 8 for *ttbk2* dKO. Error bars represent the mean ± SD. *p < 0.05 by Student’s t test.

To corroborate these observations in vivo, we manipulated TTBK2 expression in GNPs within the EGL of the developing mouse cerebellum. Plasmids encoding sh*Ttbk2* or shCtrl, along with GFP, were electroporated at P6, and the GFP-labeled cells were examined 2 days later, by which time some GNPs had initiated differentiation. Cerebellar sections were stained with Ki67 to assess the effects of Ttbk2 knockdown on GNP proliferation. In cerebella electroporated with shCtrl, approximately 60% of GFP+ cells were still actively proliferating in the EGL. In contrast, only ~30% of GFP+ cells remained in the proliferating state of the sh*Trbk2*-electroporated group (Fig. 2A–C), confirming that Ttbk2 inactivation leads to premature GNP differentiation. Meanwhile, we observed that the ciliated frequency was dramatically lower for sh*Ttbk2*-electroporated cerebella, suggesting that Ttbk2 knockdown destabilizes primary cilia (Figs. 2D and S2A).

We also electroporated TTBK2 cDNA into GNPs in the EGL at P6 and examined their proliferative state 2 days later. We observed that more GFP+ cells remained in the EGL and were Ki67+ for the cerebella electroporated with TTBK2 than the case for control brains electroporated with GFP alone, indicating that elevated TTBK2 levels can maintain GNPs in a proliferative state (Fig. 2E, F). To examine if TTBK2 kinase activity is required for its function in GNP proliferation, we mutated TTBK2 residue D163 from aspartic acid to alanine (TTBK2^KD^) [24, 26] and expressed TTBK2^KD^ in GNPs. Surprisingly, a majority of TTBK2^KD^-expressing cells exited the cell cycle and migrated prematurely to the IGL, indicating that TTBK2-dependent GNP proliferation relies on TTBK2 activity (Fig. 2E, F). By performing Arl13b staining, TTBK2 overexpression in GNPs increased the percentage of ciliated GNPs, indicating that elevated TTBK2 levels in GNPs stabilize primary cilia (Figs. 2G and S2B). Furthermore, in developing cerebella electroporated with TTBK2^KD^, proportions of ciliated GNPs were greatly reduced (Figs. 2G and S2B), confirming that TTBK2 supports GNP proliferation through TTBK2’s activity-dependent stabilization of primary cilia.

### Disruption of *ttbk2* reduces granule precursor pools in zebrafish

We generated *ttbk2* knockout zebrafish to further confirm the role of TTBK2 in GNP proliferation. TTBK2 is highly conserved across vertebrates, from zebrafish to humans [36]. Zebrafish possess two orthologous *ttbk2* genes, namely *ttbk2a* and *ttbk2b* (Fig. 2H). We disrupted the *ttbk2* genes by introducing mutations in both *ttbk2a* and *ttbk2b* (Fig. 2H). Crossbreeding of *ttbk2a*^*+/*−^ and *ttbk2b*^*+/−*^ fish yielded complete dual knockout (dKO) fish (*ttbk2a*^*-/-*^; *ttbk2b*^*-/-*^), referred to as *ttbk2* dKO (Fig. 2I).

We examined cerebellar development in these *ttbk2* dKO zebrafish, with normal *ttbk2*-harboring siblings serving as controls (CTR). The *ttbk2* dKO zebrafish were unable to swim and died shortly after birth, indicating cerebellar dysfunction in the absence of *ttbk2* (Fig. S2C). We then analyzed the expression of *atoh1a* in the upper rhombic lip (URL) of zebrafish hindbrains to identify GNPs [37, 38]. For both CTR and *ttbk2* dKO zebrafish, GNPs were detected through in situ hybridization of *atoh1a* at 2 days post-fertilization (Fig. 2J). By quantifying the volume of the *atoh1a*-expressing region, the GNP domain of *ttbk2* dKO fish was significantly reduced compared to CTR fish, confirming that ttbk2 disruption in zebrafish reduces GNP pools (Fig. 2J, K).

### TTBK2 stabilizes primary cilia

**Fig. 3:**
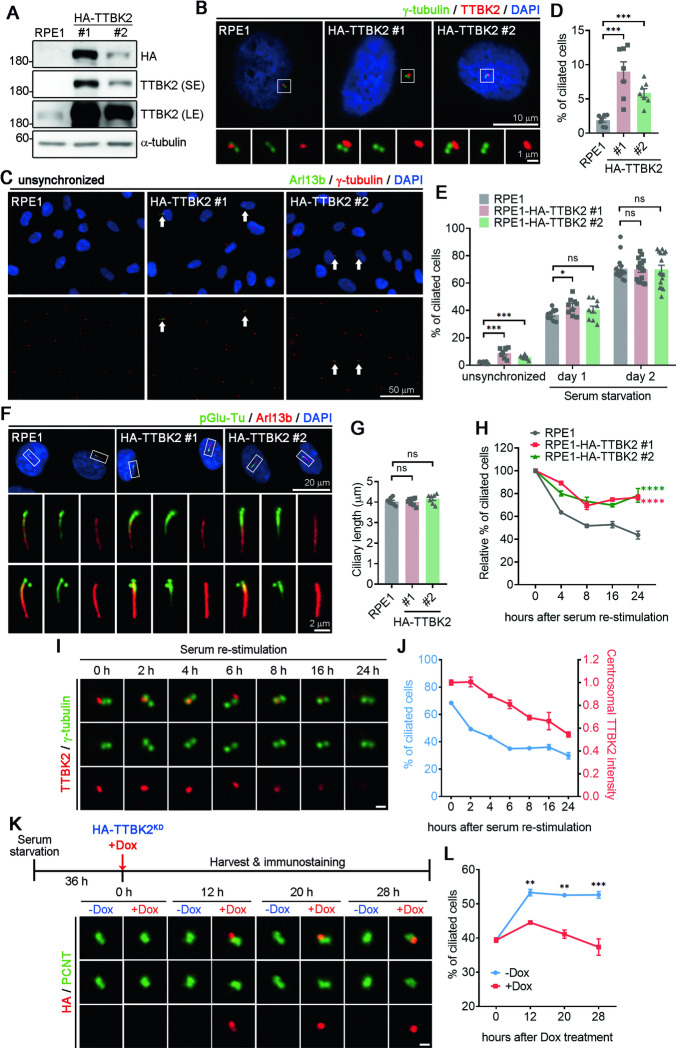
The elevated TTBK2 expression stabilizes primary cilia. **A** HA-TTBK2 was stably expressed in RPE1 cells. Two TTBK2-expressing cell lines (#1 and #2) were selected. TTBK2 levels were examined by immunoblots using antibodies as indicated. SE: short exposure. LE: long exposure. **B** Immunostaining was performed in the control and two TTBK2-overexpressing RPE1 cells using antibodies as indicated. Nuclei were stained by DAPI. Regions within the marked boxes were magnified and shown in the bottom. Scale bars are as indicated. **C** RPE1 cells cultured without SHH were fixed and stained with antibodies against Arl13b and γ-tubulin. Nuclei were stained by DAPI (blue). The arrows in the images highlight the presence of primary cilia. Scale bars are as indicated. **D**, **E** The percentage of ciliated cells was quantified in unsynchronized and serum-starved cells. More than 200 cells from at least 7 independent experiments were tested. Error bars represent the mean ± SEM. ns not significant, ***p < 0.001, *p < 0.05 by Student’s t test. **F** Immunostaining was conducted in cells that had been serum-starved for 2 days, using antibodies against glutamylated tubulin and Arl13b. Scale bars are as indicated. **G** Ciliary length was quantified by measuring more than 200 cells from at least 7 independent experiments. Error bars represent the mean ± SEM. ns, not significant by Student’s t test. **H** Cells were serum-starved for two days before adding serum to induce primary cilia disassembly. Ciliated frequency was determined using Arl13b staining. More than 200 cells from n = 3 independent experiments were tested. Error bars represent the mean ± SEM. ****p < 0.0001 by Two-way ANOVA. **I** Immunostaining was performed with antibodies against TTBK2 (red) and γ-tubulin (green). Scale bar, 1 μm. **J** The percentage of ciliated cells and TTBK2 intensity at the centrosomes were quantified. At least 200 cells from n = 3 independent experiments were tested. Error bars represent the mean ± SEM. **K** Experimental timeline schematic for assaying primary cilia in doxycycline-inducible HA-TTBK2^KD^ RPE1 cells is shown. Immunostaining was performed with antibodies against HA (red) and PCNT (green). Representative images were shown. Scale bar, 1 μm. **L** The percentage of ciliated cells was quantified. More than 200 cells were analyzed for each independent experiment. Error bars represent mean ± SEM. n = 3. **p < 0.01, ***p < 0.001 by Student’s t test.

We generated RPE1 cells overexpressing HA-tagged TTBK2 (HA-TTBK2) to investigate how TTBK2 regulates primary cilia stability (Fig. 3A). Immunostaining of TTBK2 confirmed the increase of TTBK2 signals at the centrosome (Fig. 3B). Examination of primary cilia revealed a significant increase in the ciliated frequency for two TTBK2-overexpressing cell lines (Fig. 3C, D). To determine if this effect arose from changes in cell cycle progression, we performed flow cytometry and detected no significant difference in cell cycle progression between control cells and TTBK2-overexpressing cells (Fig. S3). To investigate if elevated TTBK2 expression promoted ciliogenesis or extended cilia length, cells were subjected to serum starvation to induce cilia formation, but neither cilia formation nor cilia length was affected by TTBK2 overexpression (Fig. 3E–G), indicating that the increased frequency of ciliation by TTBK2 overexpression was not attributable to a promotion of ciliogenesis or cilia elongation.

Next, we investigated the effect of elevated TTBK2 expression on primary cilia disassembly. Cells were serum-starved for two days and then reintroduced serum to induce cilia disassembly, with the ciliated frequency being determined at 2, 4, 6, 8, 16, and 24 h after serum reintroduction. In control cells, the percentage of ciliated cells fell to ~40% 24 h after serum reintroduction. Remarkably, TTBK2-overexpressing cell presented a significantly slower rate of cilia disassembly over time compared to control cells, indicating that elevated TTBK2 levels prevent primary cilia disassembly (Fig. 3H). We also detected a reduction for TTBK2 signals at centrosomes over time during primary cilia disassembly (Fig. 3I, J), further supporting that TTBK2 stabilizes primary cilia by preventing primary cilia disassembly.

To know if TTBK2 kinase activity is required for its function in stabilizing primary cilia, we established a RPE1 cell line expressing HA-TTBK2^KD^ under a doxycycline (Dox)-inducible promoter. Cells were serum starved to induce cilia formation and then added Dox to allow HA-TTBK2^KD^ expression (Fig. 3K). Dox treatment induced HA-TTBK2^KD^ expression at the centrioles, as verified by HA staining (Fig. 3K). Interestingly, expression of TTBK2^KD^ elicited primary cilia disassembly in serum-starved cells, indicating a necessity for TTBK2 kinase activity in stabilizing primary cilia (Fig. 3L).

### Regulation of TTBK2 levels in GNPs through ubiquitin-dependent proteolysis

**Fig. 4:**
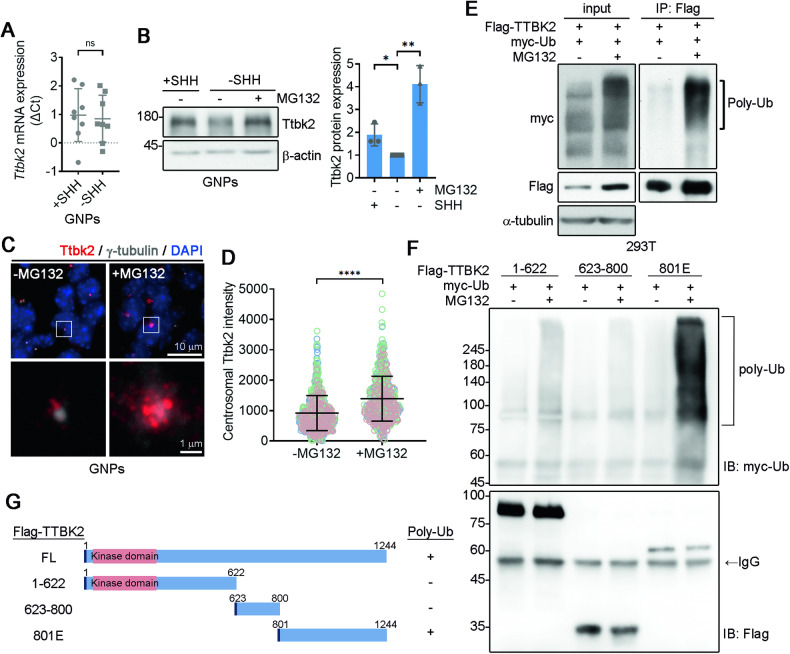
TTBK2 level in GNPs is controlled by ubiquitin-dependent proteolysis. **A** *Ttbk2* expression was examined by qPCR analysis in purified P7 GNPs cultured with or without SHH ligands for 3 days. Error bars represent mean ± SEM; n = 8. ns, not significant by Student’s t test. **B** Purified P7 GNPs were treated with or without SHH for 2 days. In the absence of SHH ligand for 36 h, GNPs were treated with MG132 (5 μM) for another 8 h. Immunoblots were performed and the Ttbk2 levels were quantified. Data were collected from three independent experiments. Error bars represent the mean ± SD. *p < 0.05, **p < 0.01 by Student’s t test. **C** Immunostaining of Ttbk2 (red), γ-tubulin (white), and DAPI (blue)  was performed in purified GNPs treated with or without MG132. **D** The scatter plot graph shows the result of Ttbk2 intensity at centrosomes from **C** from three independent experiments. Each color represents an individual experiment. Around 200 cells were counted per experiment. Error bars represent the mean ± SD. ****p < 0.0001 by nonparametric test. **E** Ubiquitination assay using lysate from 293T cells expressing Flag-TTBK2 and myc-Ub in the absence or presence of MG132. Immunoprecipitation of TTBK2 was performed followed by immunoblots with antibodies as indicated. **F** Ubiquitination assay was performed using lysates from 293T cells expressing Flag-tagged TTBK2 deletion mutants and myc-Ub in the absence or presence of MG132. Immunoprecipitation of TTBK2 was performed followed by WBs using antibodies as indicated. **G** The ability of each TTBK2 mutant to form the high-molecular-mass of poly-Ub molecules is shown.

Here, we investigated the regulatory mechanism controlling Ttbk2 levels in the developing cerebellum. To determine if Ttbk2 expression is transcriptionally regulated, we conducted a qPCR analysis on the *Ttbk2* gene in primary GNPs cultured with or without SHH. qPCR analysis of *Ttbk2* uncovered no difference between proliferating and differentiating GNPs, indicating Ttbk2 expression in GNPs is not transcriptionally controlled (Fig. 4A). Accordingly, we turned our attention to the role of proteasome-dependent proteolysis in regulating TTBK2 levels during GNP differentiation. Treatment of MG132 in SHH-untreated GNPs significantly increased TTBK2 levels (Fig. 4B), suggesting that TTBK2 levels are regulated by proteasome-dependent proteolysis. Furthermore, Ttbk2 staining revealed strong Ttbk2 signal at the centrosomes when the GNPs were treated with MG132, further supporting that Ttbk2 levels during GNP differentiation are regulated by proteolysis (Fig. 4C, D).

Through co-expression of myc-ubiquitin and Flag-TTBK2 in 293T cells, followed by TTBK2 immunoprecipitation, we observed a dramatic accumulation of high-molecular-mass polyubiquitinated (poly-Ub) molecules in TTBK2 immunoprecipitates, indicating that TTBK2 is an ubiquitinated protein (Fig. 4E). To map the ubiquitination sites on TTBK2, we generated TTBK2 deletion mutants to perform TTBK2 ubiquitination assays (Fig. 4F, G). Among these mutants, only cells expressing TTBK2^801E^ exhibited high-molecular-mass poly-Ub molecules, indicating that the ubiquitination sites of TTBK2 are at TTBK2 C-terminus (Fig. 4F, G).

### HUWE1-mediated TTBK2 degradation negatively regulates ciliogensis

**Fig. 5:**
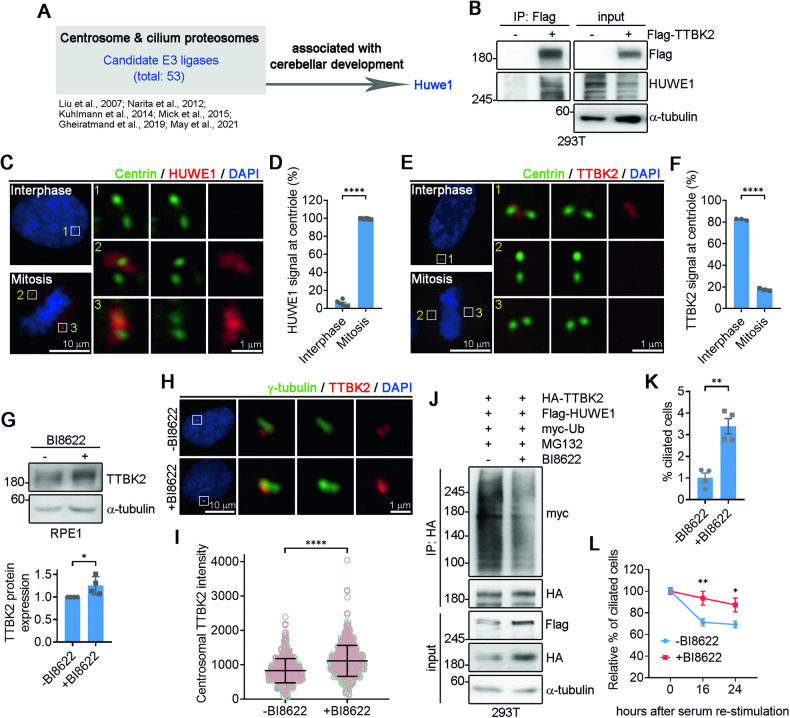
HUWE1 controls cilia disassembly through degrading TTBK2. **A** Workflow for searching candidate centrosomal E3 ligases involved in the regulation of TTBK2 degradation. **B** Flag-TTBK2 was ectopically expressed in 293 T cells. TTBK2 immunoprecipitation was performed followed by immunonblots with indicated antibodies. **C** RPE1 cells were stained with antibodies to against centrin and HUWE1. Nuclei were stained by DAPI. Regions within the marked boxes were magnified and shown in right. Scale bars are as indicated. **D** HUWE1 positive signals at centrioles were quantified. Data were collected from n = 4 independent experiments. Error bars represent the mean ± SEM. ****p < 0.0001 by Student’s t test. **E** RPE1 cells were stained with antibodies to against centrin and TTBK2. Nuclei were stained by DAPI. Regions within the marked boxes were magnified in right. Scale bars are as indicated. **F** TTBK2 positive signals at centrosome were quantified. Data were collected from n = 3 independent experiments. Error bars represent the mean ± SEM. ****p < 0.0001 by Student’s t test. **G** RPE1 cells were treated with BI8622 for 24 h. WB analysis was performed using antibodies as indicated with α-tubulin as the loading control. TTBK2 levels were quantified. Data were collected from n = 4 independent experiments. Error bars represent the mean ± SD. *p < 0.05 by Student’s t test. **H** Cells were treated with BI8622 for 24 h followed by immunostaining with anti-TTBK2 and anti-γ-tubulin antibodies. Nuclei were stained by DAPI. Regions within the marked boxes were magnified in the right. Scale bars are as indicated. **I** The TTBK2 intensity at the centrosome was quantified. The scatter plot graph showed the results from three experiments. Each color represents an individual experiment. More than 200 cells were counted per experiment. Error bars represent the mean ± SD. ****p < 0.0001 by nonparametric test. **J** Ubiquitination assay was carried out in 293 T cells that transfected with HA-TTBK2, Flag-HUWE1, and myc-Ub. Cells were treated with BI8622 for 24 h before harvesting to inhibit HUWE1 activity. Immunoprecipitation of TTBK2 was performed followed by immunoblots with antibodies as indicated. **K** RPE1 cells were treated with BI8622 for 24 h. The ciliated frequency was determined by Arl13b staining. Data were collected from n = 4 independent experiments. Error bars represent the mean ± SEM. **p < 0.01 by Student’s t test. **L** Cilia disassembly assay was performed. The ciliated frequency was quantified. More than 200 cells were analyzed for each independent experiment. Error bars represent mean ± SEM. n = 4. *p < 0.05, **p < 0.01 by Student’s t test.

We next sought to identify the E3 ubiquitin ligase responsible for TTBK2 degradation. Through analyzing the centrosome and cilia proteomes (Figs. 5A and S4) [17–22], HUWE1 emerged as a promising candidate. After expressing Flag-TTBK2 in 293 T cells, we conducted immunoprecipitation using anti-Flag antibody. We confirmed an interaction between TTBK2 and HUWE1, as HUWE1 was detected within the Flag-TTBK2 immunocomplexes (Fig. 5B). Additionally, HUWE1 staining showed that HUWE1 localizes at centrosomes (Fig. 5C). Interestingly, HUWE1 signals were predominantly observed at the centrosomes during mitosis, but its signals were weaker during interphase (Fig. 5C, D). In contrast, TTBK2 signals were detected at centrosomes during interphase, but were absent during mitosis (Fig. 5E, F). This inverse correlation between HUWE1 and TTBK2 signals at the centrosomes also suggested a role for HUWE1 in degrading TTBK2.

To examine the role of HUWE1 in TTBK2 degradation, we treated RPE1 cells with the HUWE1 inhibitor BI8622. Higher TTBK2 levels were observed in the BI8622-treated cells, indicating that HUWE1 controls TTBK2 degradation (Fig. 5G). TTBK2 staining further supported this observation, revealing stronger TTBK2 signals at centrosomes upon inhibiting HUWE1 (Fig. 5H, I). TTBK2 ubiquitination assay confirmed that TTBK2 is a HUWE1 substrate, with BI8622 treatment preventing HUWE1-dependent TTBK2 ubiquitination (Fig. 5J). Given the role of HUWE1 in regulating TTBK2 degradation, it appears that HUWE1 is a negative regulator of ciliogenesis. Arl13b staining showed that BI8622 treatment significantly increased ciliated frequency of RPE1 cells and reduced their rate of cilia disassembly over time, confirming that HUWE1 promotes primary cilia disassembly (Fig. 5K, L).

**Fig. 6:**
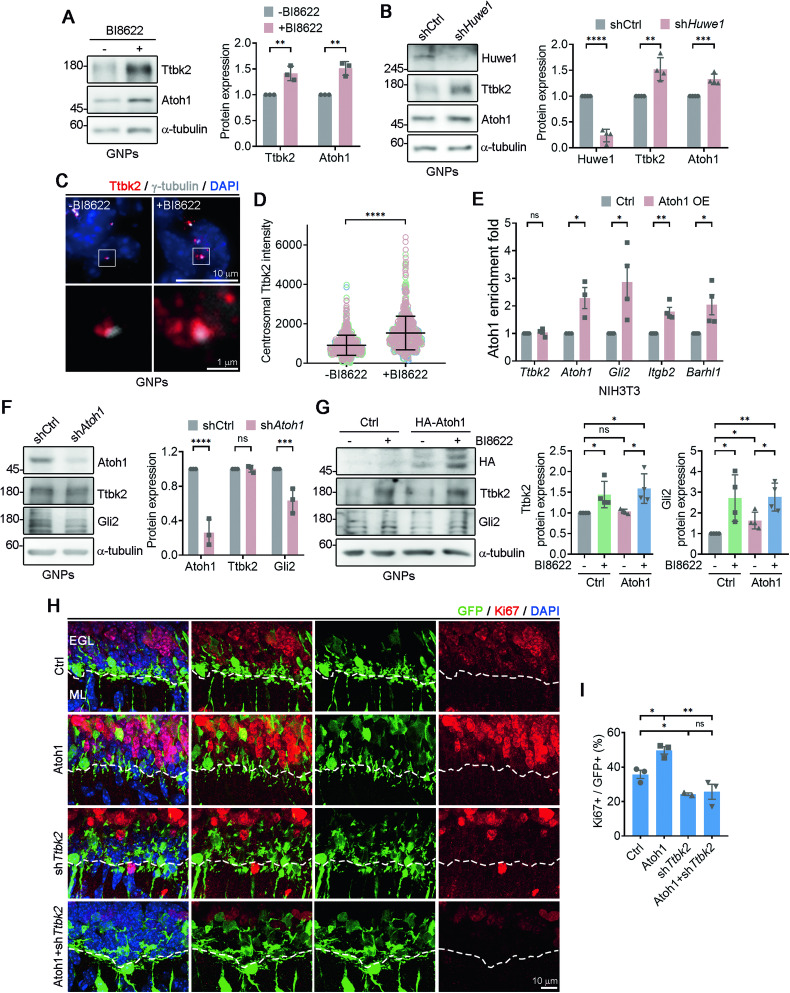
Ttbk2 degradation promoted by Huwe1 in GNPs is Atoh1 independent. **A** Purified P7 GNPs were treated with or without BI8622 for 24 h. Immunoblots were performed using antibodies as indicated. The levels of Ttbk2 and Atoh1 were quantified. Data were collected from n = 3 independent experiments. Error bars represent the mean ± SD. **p < 0.01 by Student’s t test. **B** Lentivirus carrying either shCtrl or sh*Huwe1* was used to infect purified P7 GNPs, which were then cultured for an additional 3 days. Immunoblots were performed using antibodies as indicated. Huwe1, Ttbk2 and Atoh1 levels were quantified. Data were collected from n = 4 independent experiments. Error bars represent the mean ± SD. ****p < 0.0001, ***p < 0.001, **p < 0.01 by Student’s t test. **C** Purified P7 GNPs were treated with or without BI8622 for 24 h. Immunostaining was performed using antibodies as indicated. Nuclei were stained by DAPI. Regions within the marked boxes were magnified and shown in the bottom. Scale bars are as indicated. **D** The Ttbk2 intensity around the centrosome was quantified. The scatter plot graph showed the results from n = 3. Each color represents an individual experiment. More than 200 cells were counted per experiment. Error bars represent the mean ± SD. ****p < 0.0001 by nonparametric test. **E** HA-Atoh1 was stably expressed in NIH3T3 cells (Atoh1- OE). Atoh1-ChIP-qPCR analysis was performed. The enrichment folds of Atoh1 at those genes were quantified. Error bars represent the mean ± SEM from at least three independent experiments. ns, not significant, **p < 0.01, *p < 0.05 by Student’s t test. **F** Purified GNPs were infected with lentivirus carrying sh*Atoh1*. The levels of Atoh1, Ttbk2, and Gli2 were examined and quantified by western blot analysis. Error bars represent the mean ± SD from n = 3 independent experiments. ns, not significant, ****p < 0.0001, ***p < 0.001 by Student’s t test. **G** HA-Atoh1 was expressed in purified P7 GNPs. Cells were treated with or without BI8622 (10 μM) for 24 h. Immunoblots were performed using antibodies as indicated. The relative levels of Ttbk2 and Gli2 were quantified. Data were collected from n = 4 independent experiments. Error bars represent the mean ± SD. ns, not significant, **p  <  0.01. *p < 0.05 by Student’s t test. **H** Atoh1 expression construct along with shCtrl or sh*Ttbk2* was electroporated into the EGL of P6 mice followed by a waiting period of 2 days. Dashed white line distinguishes EGL (upper) and ML (lower). Immunostaining was performed to label the proliferating GNPs (Ki67+, red) and electroporated cells (GFP+). Nuclei were stained by DAPI. Scale bar, 10 μm. **I** The percentage of Ki67+/GFP+ cells in EGL was quantified. 50–200 electroporated cells were counted (n = 3 for shCtrl, Atoh1 and Atoh1+sh*TTBK2*, n = 2 for sh*Ttbk2*). Error bars represent the mean ± SEM. ns, not significant, *p < 0.05, **p < 0.01 by Student’s t test.

To validate the role of HUWE1 in TTBK2 degradation in GNPs, we treated purified GNPs with BI8622. Ttbk2 levels were significantly higher in the BI8622-treated GNPs, indicating that Huwe1 regulates Ttbk2 stability (Fig. 6A). When we infected GNPs with lentiviruses encoding sh*Huwe1* or shCtrl, Ttbk2 levels were higher in the sh*Huwe1*-infected cells than in shCtrl-infected cells, further demonstrating that Huwe1 controls Ttbk2 stability in GNPs (Fig. 6B). Additionally, BI8622-treated GNPs presented higher Ttbk2 signals at centrosomes, providing further evidence of Huwe1’s role in regulating Ttbk2 levels in GNPs (Fig. 6C, D).

### The Ttbk2 degradation promoted by Huwe1 in GNPs is Atoh1-independent

Given that Huwe1 is known to degrade Atoh1, leading to GNP differentiation [7, 8, 16], we investigated the role of Atoh1 in Huwe1-dependent Ttbk2 degradation. Chromatin immunoprecipitation assays showed that Atoh1 did not bind to the *Ttbk2* promoter (Fig. 6E). Moreover, qPCR and Western blot analyses demonstrated that neither Atoh1 overexpression nor depletion affected Ttbk2 levels, indicating that Huwe1-dependent Ttbk2 degradation is Atoh1-independent (Figs. 6F and S5). To directly corroborate this finding, we ectopically expressed Atoh1 in isolated primary GNPs and analyzed Ttbk2 levels in cells treated with or without BI8622. We observed that BI8622 treatment increased Ttbk2 levels in both control and Atoh1-expressing cells, providing compelling evidence that Atoh1 is not involved in Huwe1’s activity in regulating Ttbk2 degradation (Fig. 6G).

To confirm in vivo that HUWE1-mediated TTBK2 degradation is independent of Atoh1, we introduced Atoh1 cDNA along with either shCtrl or sh*Ttbk2* into GNPs of the mouse EGL. Whereas ~50% of GFP+ cells were observed as actively proliferating in the EGL upon overexpressing Atoh1 [11], co-transfecting sh*Ttbk2* reduced this proportion to ~20% (Fig. 6H, I), evidencing that HUWE1-mediated Ttbk2 degradation regulates GNP proliferation independently of Atoh1.

### TTBK2 depletion impairs SHH signaling and suppresses cell proliferation in SHH-MBs

**Fig. 7:**
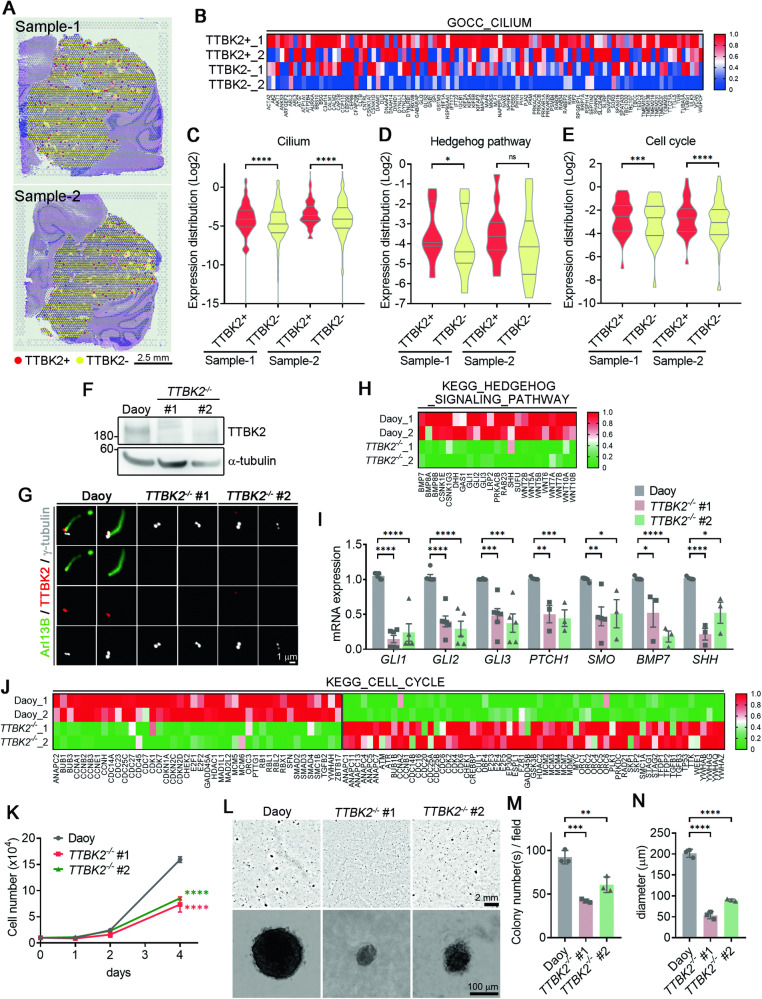
TTBK2 depletion inhibits cell proliferation of SHH-MB. **A** The putative TTBK2 clusters with respect to its spatial positions in two orthotopic xenograft SHH-MBs (sample 1 and sample 2). **B** A heatmap illustrating the gene expression levels of the top 100 genes with significant changes in TTBK2-positive and TTB2-negative groups. **C-E** Violin plots depicting the activities of cilia (in **C**), SHH (in **D**), and cell cycle (in **E**) gene signatures, separated by TTBK2 positive and TTB2 negative clusters. ns, not significant, *p < 0.05, *** p < 0.001, ****p < 0.0001 by nonparametric test. **F** Immunoblots were performed in wild type and two *TTBK2*^*-/-*^ Daoy cell lines (#1 and #2) with anti-TTBK2 and anti-α-tubulin antibodies. **G** Immunostaining was performed with antibodies as indicated. Scale bar, 1 μm. **H** Total RNAs in wild-type and *TTBK2* knockout Daoy cells were isolated for RNA-seq analysis (n = 2). The row z-score-normalized heatmap shows the down-regulated genes associated with hedgehog signaling. **I** The expression of SHH target genes was analyzed by qPCR. Data were normalized to the internal control (18S). Data were from n = 3 independent experiments. Error bars represent the mean ± SEM. ***p < 0.001, **p < 0.01 by Student’s t test. **J** The row z-score-normalized heatmap shows the altered genes associated with cell cycle. **K** Cell proliferation assay was performed in wild type and two *TTBK2*^*-/-*^ Daoy cell lines. Data were collected from n = 3 independent experiments. Error bars represent the mean ± SEM. ****p < 0.0001 by Two-way ANOVA. **L** The soft agar assays were performed on wild type and two *TTBK2*^*-/-*^ Daoy cells. The resulting colonies were observed under phase-contrast microscopy. Scale bars as indicated. **M** The number of colonies per field (2.3 cm^2^) was quantified. Data were collected from n = 3 experiments. Error bars represent the mean ± SD. ***p < 0.001, **p < 0.01 by Student’s t test. **N** Colony diameters were measured. More than 50 colonies were counted per experiment. Data were collected from n = 3 experiments. Error bars represent the mean ± SD. ****p < 0.0001 by Student’s t test.

The pathogenesis of SHH-MB is closely associated with uncontrolled GNP proliferation [34]. Given TTBK2’s crucial role in stabilizing primary cilia and supporting GNP proliferation, we explored its potential involvement in SHH-MB. Utilizing two spatially resolved transcriptomics from SHH patient-derived orthotopic xenograft medulloblastomas (PDOX MBs) [39], we investigated the link between TTBK2 expression and the molecular characteristics of SHH-MB. PDOX MBs were separated into TTBK2-positive and -negative clusters (Fig. 7A). Gene expression uncovered significant upregulation of ciliogenesis and cilia-related signaling in TTBK2-positive cluster (Fig. 7B). Differential gene expression analysis of the TTBK2-postive and -negative clusters showed the positive correlation between TTBK2 expression and genes related to cilia, HH signaling, and the cell cycle, indicating that TTBK2 supports cell proliferation in SHH-MBs by stabilizing primary cilia (Fig. 7C–E).

To investigate the therapeutic potential of TTBK2 in SHH-MB, we generated *TTBK2* knockout (*TTBK2*^*-/-*^) Daoy cells (Figs. 7F, G and S6). RNA-sequencing analysis of wild-type and *TTBK2* knockout Daoy cells revealed that TTBK2 depletion downregulated genes associated with Hedgehog signaling (Fig. 7H). qPCR analysis further confirmed that TTBK2 depletion reduced the expression of SHH target genes (Fig. 7I). Notably, TTBK2 depletion also altered for genes linked to the cell cycle (Fig. 7J). Subsequent cell proliferation and clonogenic assays demonstrated a significant inhibition of cell proliferation upon TTBK2 depletion (Fig. 7K–N). Cell proliferation assay of UW228.2 cells also revealed markedly reduced cell proliferation upon TTBK2 depletion, further confirming a critical role for TTBK2 in supporting SHH-MB proliferation (Fig. S7). In conclusion, our findings support the notion that TTBK2 inhibition represents a potential anti-cancer strategy for SHH-MBs.

## Discussion

**Fig. 8:**
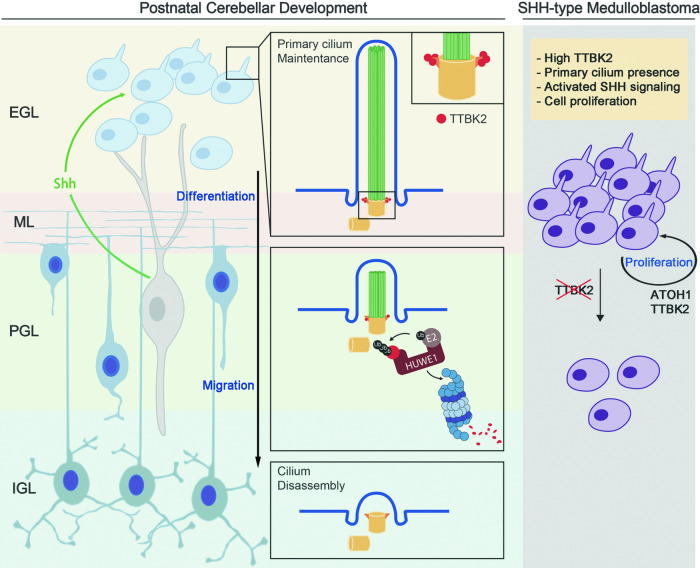
Proposed model for HUWE1-dependent TTBK2 degradation in the regulation of cerebellar development and SHH-MB growth. Our findings reveal the crucial role of SHH signaling in preventing TTBK2 degradation, which in turn stabilizes primary cilia on GNP surfaces and supports GNP proliferation. Additionally, our results suggest that TTBK2 inhibition is a potential therapeutic strategy for SHH-MB.

In this study, we have investigated the mechanisms governing GNP proliferation and their implications for cerebellar development and medulloblastoma. Our findings reveal a complex regulatory network centered on TTBK2 and HUWE1. We elucidate a crucial role for HUWE1 in cerebellar development, particularly in terms of promoting GNP differentiation by regulating primary cilia. Furthermore, our findings underscore the significance of SHH signaling in preventing TTBK2 degradation, thereby stabilizing primary cilia on GNP surfaces to support GNP proliferation. Our results also highlight TTBK2 as a potential therapeutic target for this specific type of brain tumor (Fig. 8).

Our study has revealed that HUWE1-dependent TTBK2 degradation acts independent of Atoh1 [7, 8, 11, 16]. This scenario raises intriguing questions about how Ttbk2 levels are maintained in proliferating GNPs. It could be assumed that Atoh1 maintains Ttbk2 levels, given that Atoh1 orchestrates GNP proliferation. However, our results have shown that neither overexpression nor depletion of Atoh1 affects Ttbk2 levels (Figs. 6F, G and S5). Since SHH signaling is required for GNP proliferation [4, 11, 40, 41], it is possible that *Ttbk2* is one of the gene targets of SHH signaling. The SHH modulates target gene expression via Gli transcription factors. It would be interesting to investigate if any of these factors bind to the *Ttbk2* promoter and strongly elevate its high expression in proliferating GNPs. Our findings also indicate the presence of multiple parallel pathways and regulators orchestrating GNP differentiation, underscoring the intricate nature of this process.

We have identified HUWE1 as an E3 ubiquitin ligase responsible for controlling TTBK2 levels in GNPs. HUWE1 negatively regulates ciliogenesis by targeting TTBK2 for ubiquitination and subsequent degradation. Our experiments involving HUWE1 inhibition, both in vitro and in GNPs, consistently resulted in elevated TTBK2 levels, emphasizing the role of HUWE1 in fine-tuning TTBK2 stability. Given the link between uncontrolled GNP proliferation and SHH-MB, our findings illuminate TTBK2 as a potential therapeutic target for this specific brain tumor subtype. HUWE1 has also been identified as a potential therapeutic target in brain cancer due to its underexpression in brain tumors [16, 42, 43]. Previous studies have also shown that low HUWE1 expression is associated with a poor prognosis, specifically within SHH-MB. Our results demonstrate that TTBK2 depletion significantly reduces cell proliferation in SHH-MB cell lines (Fig. 7K–N, and Fig. S7). It should prompt further investigations to explore the therapeutic efficacy of targeting TTBK2 and its downstream signaling pathways.

Although we have confirmed the interaction between HUWE1 and TTBK2 (Fig. 5B), the underlying mechanism by which HUWE1 recognizes and induces TTBK2 degradation remains elusive. In many cases, substrate phosphorylation serves as a signal for E3 ligase recognition and ubiquitination [44]. For example, SHH signal maintains Atoh1 levels by preventing Atoh1 phosphorylation. In the absence of SHH, Atoh1 phosphorylation at Ser328 and Ser339 recruits Huwe1, leading to Atoh1 degradation [16]. Therefore, it would be valuable to identify the phosphorylation sites on Ttbk2 that recruit Huwe1, thereby causing Ttbk2 degradation.

It is worth noting that ubiquitination typically occurs at specific lysine residues of a protein. We have show herein that the Huwe1-depepndent Ttbk2 ubiquitination site(s) are located at TTBK2 C-terminus (Fig. 4G). Given that there are 34 lysine residues in this region, it is likely that multiple ubiquitination sites exist on TTBK2. Identification of the ubiquitination sites on TTBK2 will be an important step in understanding the mechanism by which Huwe1 recognizes TTBK2 for ubiquitination.

In conclusion, our work highlights the intricate interplay between TTBK2, HUWE1, and primary cilia in regulating GNP proliferation during cerebellar development. Furthermore, our findings suggest a potential therapeutic strategy for SHH-MB by targeting TTBK2. These insights advance our understanding of cerebellar development and provide new directions for the development of effective treatments for cerebellar disorders and MB.

## Supplementary information

Supplementary information

Uncropped original blots

## Data Availability

Data in the main text or the supplementary information are available from the corresponding authors on reasonable request. The RNA-seq data generated from this study has been deposited under accession number GSE268621 in the National Center for Biotechnology Information Gene Expression Omnibus database.
