# Supplementary material for: Regulation of primary cilia disassembly through HUWE1-mediated TTBK2 degradation plays a crucial role in cerebellar development and medulloblastoma growth
Source: Cell Death Differ. 2024 Jun 15;31(10):1349–61. doi: 10.1038/s41418-024-01325-2 (PMC11445238; doi:10.1038/s41418-024-01325-2)
Supplement: Supplementary file 1 — Supplementary information [file 41418_2024_1325_MOESM1_ESM.docx]

**SUPPLEMENTARY INFORMATION**

**Regulation of Primary Cilia Disassembly Through HUWE1-Mediated TTBK2 Degradation Plays a Crucial Role in Cerebellar Development and Medulloblastoma Growth**

I-Hsuan Lin^1,2^, Yue-Ru Li^3^, Chia-Hsiang Chang^3^, Yu-Wen Cheng^2^, Yu-Ting Wang^4,5^, Yu-Shuen Tsai^6^, Pei-Yi Lin^4,5^, Chien-Han Kao^2^, Ting-Yu Su^2^, Chih-Sin Hsu^6^, Chien-Yi Tung^6^, Pang-Hung Hsu^7^, Olivier Ayrault^8,9^, Bon-chu Chung^4,10^, Jin-Wu Tsai^1,3,11,12*^, Won-Jing Wang^1,2,11*^

^1^Taiwan International Graduate Program in Molecular Medicine, National Yang Ming Chiao Tung University and Academia Sinica, Taipei, Taiwan

^2^Institute of Biochemistry and Molecular Biology, National Yang Ming Chiao Tung University, Taipei, 112, Taiwan

^3^Institute of Brain Science, School of Medicine, National Yang Ming Chiao Tung University, Taipei, 112, Taiwan

^4^Institute of Molecular Biology, Academia Sinica, Taipei, Taiwan

^5^Department of Life Sciences, National Central University, Taoyuan, 300, Taiwan

^6^Cancer and Immunology Research Center, National Yang Ming Chiao Tung University, Taipei, 112, Taiwan

^7^Department of Bioscience and Biotechnology, National Taiwan Ocean University, Keelung, 20224, Taiwan

^8^Institut Curie, PSL Research University, CNRS UMR, INSERM, Orsay, France

^9^Université Paris Sud, Université Paris-Saclay, CNRS UMR, INSERM U, Orsay, France

^10^Graduate Institute of Biomedical Sciences, Neuroscience and Brain Disease Center, China Medical University, Taichung, 404, Taiwan
^11^Advanced Therapeutics Research Center, National Yang Ming Chiao Tung University, Taipei, 112, Taiwan
^12^Brain Research Center, National Yang Ming Chiao Tung University, Taipei, 112, Taiwan

*Correspondence should be address to: J.W.T. ([tsaijw@nycu.edu.tw](file:///C:\Users\ihsua\Desktop\Thesis%20STUDY%20and%20Writing\draft_Dev%20cell%20format\tsaijw@nycu.edu.tw)); W.J.W. ([wangwj@nycu.edu.tw](file:///C:\Users\ihsua\Desktop\Thesis%20STUDY%20and%20Writing\draft_Dev%20cell%20format\wangwj@nycu.edu.tw))

**SUPPLEMENTARY MATERIALS AND METHODS**

**Cell culture and reagents**

293T (ATCC, CRL-3216), 293FT (Thermo Fisher Scientific, R70007), NIH3T3 and Daoy cells were cultured in Dulbecco’s Modified Eagle’s Medium (DMEM; Thermo Fisher Scientific, 12800017) supplemented with 10% fetal bovine serum and 1% penicillin-streptomycin (Thermo Fisher Scientific, 15140163). UW228.2 cells were kindly provided by Olivier Ayrault and cultured in DMEM supplemented with 10% fetal bovine serum, 1% penicillin-streptomycin (Thermo Fisher Scientific, 15140163), and 1mM sodium pyruvate (Corning, 25-000-CI). NIH3T3 cells were kindly provided by Hong-Chen Chen (National Yang Ming Chiao Tung university). RPE1 (hTERT-RPE1; ATCC, CRL-4000) cells were cultured in DMEM/F-12 (1:1) medium (Thermo Fisher Scientific, 12400024) supplemented with 10% fetal bovine serum and 1% penicillin-streptomycin. The HUWE1 inhibitor BI8622 (HY-120929) was purchased from MedChemExpress.

**Primary antibodies**

Primary antibodies were obtained from the following sources and used according to the manufacturers’ instructions: rabbit anti-TTBK2 (immunofluorescence (IF) 1:2000; western blot (WB) 1:1000; Atlas Antibodies, HPA018113), mouse anti-Centrin (IF 1:1000; Millipore, 04-1624), mouse-anti-Arl13b (IF 1:1000; Abcam, ab1336648 for RPE1; NeuroMab, 75-287 for GNPs, Daoy, UW228.2 & cerebellar slices), mouse anti-γ-tubulin (IF 1:1000; Santa Cruz, sc-51715 for RPE1 & GNPs; Abcam, ab11317 for cerebellar slices), rabbit anti-Pericentrin (PCNT; IF 1:2000; Abcam, ab4448), mouse anti-poly-Glutamylated tubulin (IF 1:3000; Adipogen, AG-20B-0020-C100), mouse anti-HA (IF 1:1000; WB 1:1000; Convance, MMS-101P), chicken anti-GFP (IF 1:2000; Abcam, ab13970), rabbit anti-Cyclin A (IF 1:1000; Thermo Fisher Scientific, PA5-34682), raddit anti-Ki67 (IF 1:500; Abcam, ab15580), rabbit anti-Myc (WB 1:1000; Cell signaling, #2278), mouse anti-Flag (WB 1:5000; Sigma-Aldrich, F3165), rabbit anti-Huwe1 (WB 1:1000; Proteintech, 19430-1-AP), rabbit anti-Atoh1 (WB 1:1000; Proteintech 21215-1-AP), rabbit anti-Gli2 (WB 1:1000; Proteintech, 18989-1-AP), mouse anti-β-actin (WB 1:1000; Novus, NB600-501), mouse anti-α-tubulin (WB 1:5000; Sigma-Aldrich, T6199).

**Isolation and culture of granule neuron progenitors**

GNPs were purified from 7-day-old (P7) mouse cerebella by Percoll gradient separation as previously described ^1, 2, 3^. Briefly, cerebella were digested in solution containing papain (20 units/ml; Sigma-Aldrich, P4762) and DNase I (100 units/ml; BioShop, DRB001) followed by triturating to obtain a cell suspension. This suspension was then centrifuged through 35% and 60% Percoll (Sigma-Aldrich, P1644). The GNPs were harvested from the 35% and 60% interface. The purified GNPs were grown on coverslip or dishes that coated with 5% Matrigel (Corning, 354234) and cultured in Neurobasal medium (Thermo Fisher Scientific, 21103049) supplemented with B27 supplement (Thermo Fisher Scientific, 17504044), 2 mM L-glutamine (Thermo Fisher Scientific, 25030081), 1 % penicillin-streptomycin (Thermo Fisher Scientific, 15140163), 0.45% D-glucose (Sigma-Aldrich, G8769), 1x linoletic acid-oleic acid-albumin (Sigma-Aldrich, L9655), and 1x SPITE medium supplement (Sigma-Aldrich, S5666). SHH-N recombinant protein (1 μg/ml; R&D system, 461-SH) or SHH-conditioned medium was added in the culture medium to maintain GNPs in proliferating status. To obtain SHH-conditioned medium, 293T cells (5x10^6^) were seeded on 100-mm dish and transfected with 15 μg pcDNA3.1-ShhN plasmid. The supernatants were collected 60 h after transfection followed by centrifuging at 4,000 rpm for 10 min and passing through 0.45 μm filter to remove cell debris (Sartorius, 16555-K).

**Cerebellar electroporation *in vivo***

P6 mouse pups were anesthetized on ice for 2 min until they had no response to pain stimuli. An incision (~1 cm) through the occipital skin between two ears was made to expose the skull above the cerebellum. A tiny hole (1 mm) in the skull below the bottom edge of the triangular midbrain surrounded the vessel was made by 26-gauge needle. A 33-gauge needle was inserted into the primary fissure of cerebellum to deliver plasmid DNA mixing with 10% Fast Green. After injection, a tweezer-type electrode, which both tips of the electrodes represent positive, was attached to mice head behind two ears and connected to a square wave generator (ECM 830, Harvard Apparatus). The square wave generator provided 6 short 70 volt (V) current pluses that the duration was 50 ms and the interval was 150 ms for cerebellar electroporation. Then, the wound was sutured and disinfected with 70% alcohol. Certain days after the surgery, cerebellum was collected through perfusion.

**Animal husbandry**

Pregnant ICR mice were purchased from BioLASCO Taiwan Co., Ltd. The offspring at various postnatal ages were used for western blot analysis, GNP culture or *in vivo* electroporation. The care and use of animals in this study were approved by the Institutional Animal Care and Use Committee (IACUC) at National Yang Ming Chiao Tung University. Zebrafish were reared at 28.5 °C under 14 h:10 h light-dark cycle. All animal work was approved by the Institutional Animal Care and Utilization Committee of Academia Sinica.

**Plasmids**

Full-length and deletion mutants of human TTBK2 were PCR amplified and cloned into the pCDNA3.1 vector that fused with Flag and HA tag at its N-terminus (pcDNA3-FH vector). The kinase-dead mutant of TTBK2 (TTBK2^KD^) was generated using site-directed mutagenesis. The pcDNA3-FH-TTBK2 was used to establish the stably TTBK2-overexpressing RPE1 cells. For cerebellar electroporation in vivo, fragments of human TTBK2 or its kinase-dead mutant (D163A) were cloned into the bicistronic IRES-GFP expression pCIG2 vector (pCIG2-TTBK2^WT^ and pCIG2-TTBK2^KD^). pDARMO-CMVT-FLAG-HUWE1 was a gift from Eric Fischer (Addgene plasmid # 187121 ; <http://n2t.net/addgene:187121> ; RRID:Addgene_187121) ^4^. The shRNAs and bicistronic GFP expression pLKO.TRC011 and pLAS3w.eGFP vectors were obtained from the RNAi Core in Academia Sinica (<http://rnai.genmed.sinica.edu.tw/index>). The shRNAs were also cloned into pLKO.TRC011 vector for expressing in primary GNPs. The targeting sequences of shRNAs are as followed:

sh*Ttbk2*: 5’-CCAGCTTCTAACATCCGTGTT-3’

sh*Huwe1*: 5’-CCAGCTTCTAACATCCGTGTT-3’

sh*Atoh1*: 5’-CAGATGGCCCAGATCTACATC-3’

The pcDNA3.1-ShhN was used to produce ShhN-conditioned medium and was a gift from Philip Beachy (Addgene plasmid # 37680; <http://n2t.net/addgene:37680>; RRID: Addgene_37680). Mouse Atoh1 cDNA was cloned into pLAS3w.eGFP vectors. The pLAS3w.eGFP-FH-Atoh1 was used in primary GNPs and in the study to establish the stably Atoh1-expressing NIH3T3 cells. The plasmid (pRK5-ubiquitin-myc) that expressed myc-tagged ubiquitin was kindly provided by Ruey-Hwa Chen (Academia Sinica). Various deletion mutants of TTBK2 were cloned into pcDNA3-FH vector and co-expressed with pRK5-ubiquitin-myc for ubiquitination assay. Zebrafish atoh1a cDNA was a gift from Dr. Masahiko Hibi ^5^.

**Immunostaining and imaging**

RPE1, Dayo, and UW228.2 cells grown on 0.1 mg/ml poly-L-lysine-coated coverslips were fixed with methanol at -20°C for 20 min. Purified GNPs grown on 5% Matrigel-coated coverslips were fixed with 4% paraformaldehyde (PFA) for 10 min at RT followed by methanol fixation at -20°C for another 20 min. Coverslip with cells were incubated with blocking buffer (3% bovine serum albumin (BSA; wt/vol) and 0.1% Triton X-100 (Sigma-Aldrich, T9284) in PBS) for 30 min at RT. Primary antibodies were diluted in blocking buffer and incubated for 2 h at RT. Alex Fluor 488-, 594-, or 680-conjugated goat secondary antibodies (1:500; Molecular Probes) were incubated for 1 h at RT. DNA was labelled by 4′,6-diamidino-2-phenylindole (DAPI; Molecular Probes). Coverslips were mounted on slices with ProLong Diamond Antifade Mountant medium (Thermo Fisher Scientific, P36961). Fluorescent images were acquired on an upright microscope (Carl Zeiss, Axio Imager M2 ApoTome2 system) with a Plan-NEOFLUAR 63x (1.4 NA) or 100x (1.3 NA) oil-immersion objective and an Axiocam 702 mono charge-coupled device camera. Images were processed by ZEN software (Carl Zeiss).

The imaging for electroporated cerebellar slices were described previously ^3^. Briefly, mouse pups were anesthetized and perfused with Phosphate buffered saline (PBS) and 4% PFA through cardiac ventricles for fixation. Sagittal section of cerebellum was taken every 100 μm with Vibratome (Leica VT1000 S). Antigen retrieval was performed by citrate buffer (100 μM citrate with 0.1% Triton X-100 in PBS) in boiling water for 10 min. After washing with PBS, the slices were incubated in blocking buffer (10% normal goat serum (NGS) and 5% BSA in PBST) for at least 1 h at RT. Primary antibodies were diluted in blocking buffer and incubated for 2 days at 4°C. Fluorescence-conjugates secondary antibodies (1:500) were incubated for 2 h at RT. Slices were stained with DAPI for 1 h at RT and mounted with VECTASHIELD Mounting Medium. Slices or cells were imaged under an inverted laser scanning confocal microscope (LSM-700, Zeiss) with a 63x objective. The excitation wavelengths were 405 nm for DAPI, 488 nm for EGFP, 555 nm for red fluorescence, and 635 nm for infrared.

**Cilia disassembly assay**

RPE1 (6x10^4^) cells grown on 12-mm coverslips were serum starved for 2 days to induce cilia formation and then 10% serum was added back to the media to induce cilia disassembly. Cells were fixed at indicated time points with methanol followed by immunostaining. The percentages of ciliated cells in each group were counted according to immunolabelling with markers of primary cilia (Arl13b) and the centrosome (γ-tubulin or PCNT).

**Ubiquitination assay**

293T cells were transfected by calcium phosphate coprecipitation with plasmids encoding Myc-tagged ubiquitin (Ub-myc) and wild-type or variants of Flag-tagged TTBK2 (Flag-TTBK2). 2 days after transfection, transfected cells were treated with 10 μM MG132 (Sigma-Aldrich, C2211) for 8 h and then lysed with lysis buffer containing 50 mM Tris-HCl (pH8.0), 150 mM NaCl, 0.5% sodium deoxycholate, 1% Nonidet P-40 and protease inhibitor. 1 mg of cell lysates were incubated with 10 μl pre-washed anti-Flag M2 magnetic beads (Sigma-Aldrich, M8823) in 1 ml lysis buffer at 4°C for 2 h under gentle rotation. Beads were washed with lysis buffer for 6 times to remove the unbound proteins. The immunoprecipitated complex were eluted by 1x SDS sample buffer and separated by SDS gels for WB analysis.

**Immunoprecipitation**

To determine the interaction between TTBK2 and HUWE1, 293T cells were transfected with pcDNA-FH-TTBK2 plasmid using T-Pro NTR II transfection reagent (T-Pro Biotechnology, JT97-N002M). 2 days after transfection, cells were washed with PBS twice and then lysed with NP-40 lysis buffer containing 50 mM Tris-HCl (pH 8.0), 150 mM NaCl, 1% Nonidet P-40, 0.5% Triton X-100, protease and phosphatase inhibitor (Roche, 04906837001). Immunoprecipitation was conducted by incubation of 1 mg total cell lysates with 20 μl pre-washed anti-Flag M2 magnetic beads at 4°C overnight under gentle rotation. The immunoprecipitated complex were eluted by SDS sample buffer and performed WB analysis.

**Generation of *TTBK2* knockout DOAY cells**

knockout cells were generated through coexpression of the Cas9 protein with TTBK2 gRNA (targeting sequence of TTBK2: 5’-GAAAATGTTGCACTGAAGG-3’ ^6^). The targeting sequence was cloned into PX459 vector. pSpCas9(BB)-2A-Puro (PX459) V2.0 was a gift from Feng Zhang (Addgene plasmid #62988 ; <http://n2t.net/addgene:62988> ; RRID:Addgene_62988) ^7^. Knockout cells were obtained through puromycin selection and clonal propagation from a single cell. The PCR products were cloned and sequenced.

**RNA extraction, cDNA synthesis, and qPCR**

Total RNA was isolated using the Quick-RNA™ Miniprep Kit (Zymo Research, R1051). Equal amounts of cDNA were synthesized using the RevertAid RT Reverse Transcription Kit (Thermo Fisher Scientific, K1691). The reverse transcription products were amplified in a reaction mixture containing SYBR™ Green PCR Master Mix (Thermo Fisher Scientific, 4309155) and 0.6 μM of each primer. The primer sequences for qPCR are listed as followed:

*Ttbk2*: 5’-CTTGGACATGCTCACCAGGG-3’; 5’-ACGATCATTTCTCCCACAGCC-3’

*Huwe1*: 5’-GAGGGCGTAAACATACAGAGAAG-3’; 5’-CGCTGCTGTGTAAAGTGGC-3’

*Atoh1*: 5’-GAGTGGGCTGAGGTAAAAGAGT-3’; 5’-GGTCGGTGCTATCCAGGAG-3’

*Gli1*: 5’-CCAAGCCAACTTTATGTCAGGG-3’; 5’-AGCCCGCTTCTTTGTTAATTTGA-3’

*Gli2*: 5’-GGGACTCTTTAGCCTCGCAG-3’; 5’-CCACAGGGTTGAGGTAGTCAT-3’

*GLI1*: 5’-AGCGTGAGCCTGAATCTGTG-3’; 5’-CAGCATGTACTGGGCTTTGAA-3’

*GLI2*: 5’-CTGCCTCCGAGAAGCAAGAAG-3’; 5’-GCATGGAATGGTGGCAAGAG-3’

*GLI3*: 5’-GAAGTGCTCCACTCGAACAGA-3’; 5’-GTGGCTGCATAGTGATTGCG-3’

*PTCH1*: 5’-CCAGAAAGTATATGCACTGGCA-3’; 5’-GTGCTCGTACATTTGCTTGGG-3’

*SMO*: 5’-CTGTCCTGCGTCATCATCTTT-3’; 5’-CCACAGCAAGGATTGCCAC-3’

*BMP7*: 5’-GGAACGCTTCGACAATGAGAC-3’; 5’-GCAGGAAGAGATCCGATTCCC-3’

*SHH*: 5’-CTCGCTGCTGGTATGCTCG-3’; 5’-ATCGCTCGGAGTTTCTGGAGA-3’

*18S*: 5’-GATATGCTCATGTGGTGTTG-3’; 5’-AATCTTCTTCAGTCGCTCCA-3’

**Protein extraction and immunoblots**

Cells and mouse cerebellar tissues were lysed by RIPA buffer (50 mM Tris-HCl, pH 8.0, 150 mM NaCl, 1% Nonidet P-40 (NP-40; Sigma-Aldrich, NP40S), 0.5% sodium deoxycholate (Sigma-Aldrich, D6750), 0.1% SDS) supplemented with protease inhibitor (Roche, 04693132001). Bradford assay (Bio-Rad, 5000006) was used to measure protein concentration. Equal amounts of proteins were separated by SDS-PAGE under reducing condition and then transferred to PVDF membranes (Millipore, IPVH00010). Membrane blocking was performed by using TBS-T (20 mM Tris-HCl, pH 7.6, 137 mM NaCl, and 0.1% Tween 20 (BioShop, TWN508)) with 5% non-fat milk for 2 h at room temperature (RT). Incubation of primary antibodies was all performed at 4°C overnight. After washing with TBS-T, membranes were incubated with HRP-conjugated goat anti-mouse IgG (1:5000; Jackson, 115-035-003) or goat anti-rabbit IgG antibodies (1:5000; Jackson, 111-035-003) for 1 h at RT. Signals were visualized with ECL substrate and detected by ImageQuant LAS 4000 (GE healthcare life sciences).

**Generation of *ttbk2* knockout zebrafish**

The gRNA targeting sites were designed online using CHOPCHOP (<https://chopchop.cbu.uib.no/>) and Benchling (<https://www.benchling.com/>). The targeting sequence was at exon 5 of *ttbk2a* (5’- TGCTCGGGAATCGGCCCA-3’) and at exon 3 of *ttbk2b* (5’- GCGAGATCTATGAAGTGC-3’). To synthesize the gRNA, we followed the protocols described in MEGAshortscript^TM^ T7 Transcription Kit (Thermo Fisher Scientific, AM1354). The 100 pg gRNA and 200 pg Cas9 protein were co-injected into 1-cell stage embryos to create the F0 *ttbk2* mutant fish. The F1 fish generated by the breeding of F0 fish with wildtype fish were genotyped and DNA of the heterozygous fish was sequenced to confirm the site of mutation. For genotyping, the following PCR primers were used: 5’-CGCTCAAGTGTCCCTATAGTG-3’ and 5’- TACTGACCGGTCTGACTTCTTG-3’ for *ttbk2a* alleles, and 5’- TGTTCCGGTCATTTGTCATTTA-3’ and 5’- TCCATCTTCAGGACTTGTTTTG-3’ for *ttbk2b* alleles, respectively. To get *ttbk2* dKO fish, male *ttbk2a^-/-^ttbk2b^-/+^* fish was mated with female *ttbk2a^+/-^ttbk2b^-/-^* fish. This scheme would produce embryos with *ttbk2a^-/+^ttbk2b^-/+^* (25%), *ttbk2a^-/+^ttbk2b^-/-^* (25%), *ttbk2a^-/-^ttbk2b^-/+^* (25%), *ttbk2a^-/-^ttbk2b^-/-^* (25%).

**In situ hybridization**

For in situ hybridization, fish embryos were incubated with the 1 ng/µl atoh1 riboprobe at 70°C overnight using a standard protocol ^8^, which includes fixation, dehydration, rehydration, proteinase K digestion, pre-hybridization, hybridization, washing, blocking, antibody incubation, endogenous alkaline phosphatase (AP) activity inhibition, equilibration and color reaction. For the color reaction, NBT/BCIP solution (Roche) was used as a substrate for alkaline phosphatase. The NBT/BCIP-stained specimens were mounted and stored in the 100% glycerol before imaging. The NBT/BCIP staining were acquired using AxioImager Z1 micrroscope with Plan Aprochromat 10X/0.45 DIC and AxioCam 506 color CCD (Zeiss). Z-stack imaging was constructed by Zeiss ZEN2 software.

**Quantitation of in situ hybridization data**

The *athoh1a*+ GNP area was quantified based on the threshold of signals using ImageJ software. The unit of images was first converted from pixel to micrometer using the tools of Analyze > Set Scale. Then the color information of images was changed to 8-bit (Image > Type). The threshold of signal was set at 94 for *atoh1a* (Image > Adjust > Threshold). Finally, the signals higher than the threshold were analyzed (Analyze > Analyze Particles).

**Cell cycle analysis**

Approximately 1x10^6^ cells per experiment condition were harvested, fixed in 70 % cold ethanol at -20°C for 30 min. Cells were washed twice with PBS and then incubated with 10 mg/ml propidium iodide (PI; Sigma, P4170) and 20 μg/ml RNase A (BioShop, RNA888) in PBS in dark at RT for 10 min. Cell cycle profiles (10000 cells) were analyzed with flow cytometer (Becton Dickinson FACSCalibur) with the FL2 detector in a linear mode. Data were analyzed using FlowJo software.

**Atoh1 Chromatin immunoprecipitation (ChIP)-qPCR**

HA-tagged Atoh1 was stably expressed in NIH3T3 cells. Cells (3x10^5^) were collected and cross-linked with 5 ml 1% PFA (Thermo Fisher Scientific, 5025997) for 15  min at RT and quenched with 10 ml 125 mM glycine for 15  min. Cell pellets were suspended in 5 ml cold PBS buffer that contained 2 mM EDTA, followed by centrifugation at 2,600 g for 5 min at 4 °C to isolate the nuclei. The pellets were lysed in 400 μl ChIP lysis buffer (10 mM EDTA, 60 mM Tris-HCl, pH 8.1, 0.1% SDS, 1% Triton X-100) supplemented with protease inhibitor. Chromatin was sonicated twice for 12 cycles (5 s on and 5 s off) on ice. 1200 μl ChIP buffer (16.7 mM Tris-HCl, pH 8.1, 167 mM NaCl, 0.01% SDS, 1.2 mM EDTA, 1.1% Triton X-100) was then added to the sonicated samples, followed by centrifugation at 14,000 g for 10 min at 4 °C. 120 μl supernatant was collected as input. The remaining supernatant was incubated with 20 μl pre-washed anti-HA agarose beads (Thermo Fisher Scientific, A2095) for 5 h at 4°C. Agarose beads were pelleted down by centrifugation at 1100 g for 5 min at 4°C. Beads bound to immunocomplexes were washed with 1ml low salt buffer (20 mM Tris-HCl, pH 8.1, 150 mM NaCl, 2 mM EDTA, 0.1% SDS, 1% Triton X-100), 1ml high salt wash buffer (20 mM Tris-HCl, pH 8.1, 500 mM NaCl, 2 mM EDTA, 0.1% SDS, 1% Triton X-100), 1ml LiCl wash buffer (10 mM Tris-HCl, pH 8.1, 250 mM LiCl, 1 mM EDTA, 1% NP-40, 1% sodium deoxycholate), and 1 ml TE buffer (10 mM Tris-HCl, pH 8.1, 1 mM EDTA). Beads were suspended in 250 μl elution buffer (10 mM Tris-HCl, pH 8.1, 1 mM EDTA, 500 mM NaCl, 25 mM DTT, 0.1% SDS) at RT for 30 min before heating at 65°C overnight for reverse cross-linking. 20 μl 1M Tris-HCl pH6.5, 10 μl 0.5 M EDTA and 2.5 μl proteinase K (20 mg/ml) were added prior incubating at 55°C for 5 h to release genomic DNA from histones. DNA was purified by the DNA purification kit (Geneaid, DFH300) and the purified DNA was used for subsequently the quantitative real-time PCR with specific primers. Primers used for Atoh1 ChIP-qPCR are listed as followed:

*Atoh1*: 5’-CAGGCTTGGAGGGATCTCAG-3’; 5’-AAGTTACTGTGTGCCGAGGA-3’

*Ttbk2*: 5’-AGCATCACAATGACAGTGCG-3’; 5’-GGCTGACATCTTTGTAACTC-3’

*Gli1*: 5’-GAGCAATTAGGAAGTTTGGG-3’; 5’-TCCCGTCTTAGAAACACTGA-3’

*Gli2*: 5’-GTTTCCACCCTGTGACATCG-3’; 5’-AGTGATCTTTCCTGCCCCTG-3’

*Gli3*: 5’-GGATGTGTCTGTGTGAGGAC-3’; 5’-GGTCAGACTCTGCTCTCCTT-3’

*Cep131*: 5’-GGTCTGTGATTGTCTTGCCT-3’; 5’-AAGCCACCTAACCATTTTAC-3’

*Itgb1*: 5’-GGCAAGTCCAAGTTTCAAGC-3’; 5’-GACAAGAAGGATCGGGTGTT-3’

*Barhl1*: 5’-CCTTGCCTGTCTTTCTTTTC-3’; 5’-GCTTACAGGTCCCAAAAGAG-3’

**Lentivirus production and infection**

293FT (4x10^6^) cells were plated on 100-mm dish the day before transfection. 6 μg VSV-G envelop plasmid, 12 μg pCMV-dR8.91 packaging plasmid and 12 μg bicistronic GFP expression lentivirus-based constructs were transfected into 293FT cells using calcium phosphate coprecipitation. The supernatant that contained viral particles was harvested 60 h after transfection. Virus containing media were centrifuged at 4000 rpm for 10 min and then passed through a 0.45 μm filter (Sartorius, 16555-K). Lentiviruses were 100X concentrated by ultracentrifugation at 25,000 rpm for 100 min at 4°C (Beckman Coulter, Optima XL-90; SW 28 Swinging-Bucket Rotor). For tittering viruses, 293T cells were infected with serial diluted viruses, and virus titers were determined according to the number of GFP-expressing cells multiplying by the dilution factors. For infection of GNPs, isolated GNPs were mixed with viruses (MOI=5), then plated together on 5 % matrigel-coated coverslips and incubated for 12 h. Respectively, 12 h and 24 h after infection, 50 % of culture medium was replaced with an equivalent volume of virus-contained medium at the same MOI. 12 h later, the virus-contained medium was replaced with fresh culture medium, and infected cells were harvested to perform western blots or immunostaining.

**Analysis of spatial transcriptomics from SHH patient-derived orthotopic xenograft MBs**

The raw sequencing data were obtained from the database (<https://www.ebi.ac.uk/ena/browser/view/PRJEB53588>). The data was further processing using defualt pipeline of SpaceRanger (v2.1.1) with a hybrid genome reference of human and mouse genomes (refdata-gex-GRCh38-and-mm10-2020-A) provided by 10x Genomics. Only spots within the tissue region were utilized for subsequent analysis. This statistical analysis was performed using LoupeBrowser (v7.0.0), wherein "specific" regions were delineated, and the "gene expression" patterns within those regions were compared.

**RNA-Seq and bioinformatics analysis.**

Total RNAs pooled from Daoy cells of wild type and *TTBK2* knockout Daoy cells were extracted by Quick-RNA™ Miniprep Kit according to manufacturer’s instructions (Zymo Research, R1051). The quantity of the RNAs for each sample was passed the FastQC quality control for high throughput sequencing data. The extracted RNA is entrusted to Biotools Company for library preparation and RNA sequencing (RNA-seq). The clean reads were analyzed using DESeq2. To analyze the differentiated gene expression in the wild-type and *TTBK2* knockout cells, the human gene sets for hedgehog (HH) signaling (56 genes) and cell cycle (125 genes) were obtained from the molecular signatures Database (MSigDB). The datasets were normalized and displayed as the heatmaps by GraphPad Prism 8.

**Cell proliferation assay**

Daoy cells (1x10^4^) grown on 12-well plated were harvested every 2 days and cell numbers were counted. Two well duplicates were performed in each independent experiment.

**Soft agar assay**

Agarose solution and 2x DMEM medium were mixed and solidified on 6-well plate to form the bottom agar at a final concentration of 0.5% agarose. The top agar, consisting of a 0.3% DMEM-agarose mixture, was mixed with 5000 cells. The culture medium was replenished every 3-4 days, and cells were incubated for 3 weeks. The resulting colonies were observed and analyzed under phase-contrast microscopy.  Colony diameters were measured using Image J software, and the colonies with a diameter greater than 5 μm were considered as positive colonies.

**Quantification of immunofluorescence and immunoblots**

To quantify fluorescent intensity with TTBK2 protein at the centrosomes, ZEN software (Zeiss) was used to analyze the images under the same setting. A circle was drawn surrounding the centrosomes to obtain the total pixel value. ImageJ software (National Institutes of Health) was used to quantify the immunoblots. A rectangle was drawn surrounding the target band and the same setting was applied to all gel bands. Results were expressed as density means ± SEM by normalizing to the control group. All the quantifications were obtained from at least three independent experiments.

**Statistical data analysis**

All data were represented as mean with SEM or SD from at least three independent experiments. Experiment samples and numbers for statistical testing are reported in the corresponding figure legends. All P-values are from Student’s t-tests for two-group comparisons, nonparametric test, or by two-way anova. (GraphPad Prism 8; ns, not significant, ****p<0.0001, ***p<0.001, **p<0.01, *p<0.05).

**SUPPLEMENTARY FIGURES**


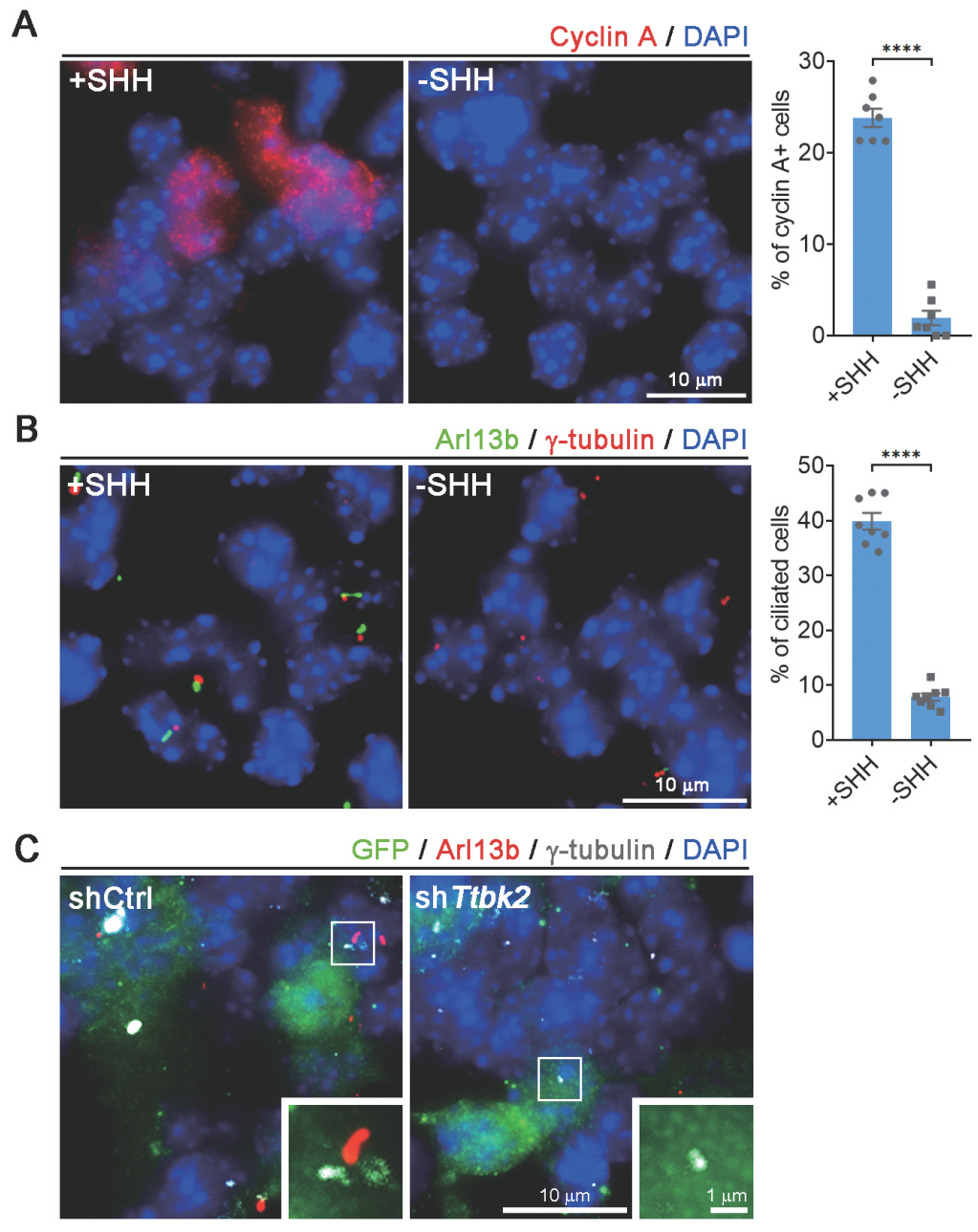


**Supplemental Fig. 1 Purify GNPs from developing cerebellum. A** Purified P7 GNPs were cultured with SHH or without SHH for 3 days. Cells were fixed and stained with the antibody against cyclin A (red). Nuclei were stained by DAPI (blue). Scale bar: 10 μm. The percentage of cyclin A positive cells was quantified. 200 cells from n=7 independent experiments were tested. Error bars represent the mean ± SEM. ****p<0.0001 by Student’s t test. **B** Cells were fixed and stained with Arl13b (green) and γ-tubulin (red). Scale bar: 10 μm. The percentage of ciliated cells was quantified. 200 cells from n=8 independent experiments were tested. Error bars represent the mean ± SEM. ****p<0.0001 by Student’s t test. **C** Purified P7 GNPs were infected with lentivirus carrying *luciferase* (shCtrl) or *Ttbk2* (sh*Ttbk2*) shRNAs along with GFP for 3 days. Cells were staining as indicated. Nuclei were stained by DAPI (blue). Regions within the marked boxes were magnified. Scale bars are as indicated.


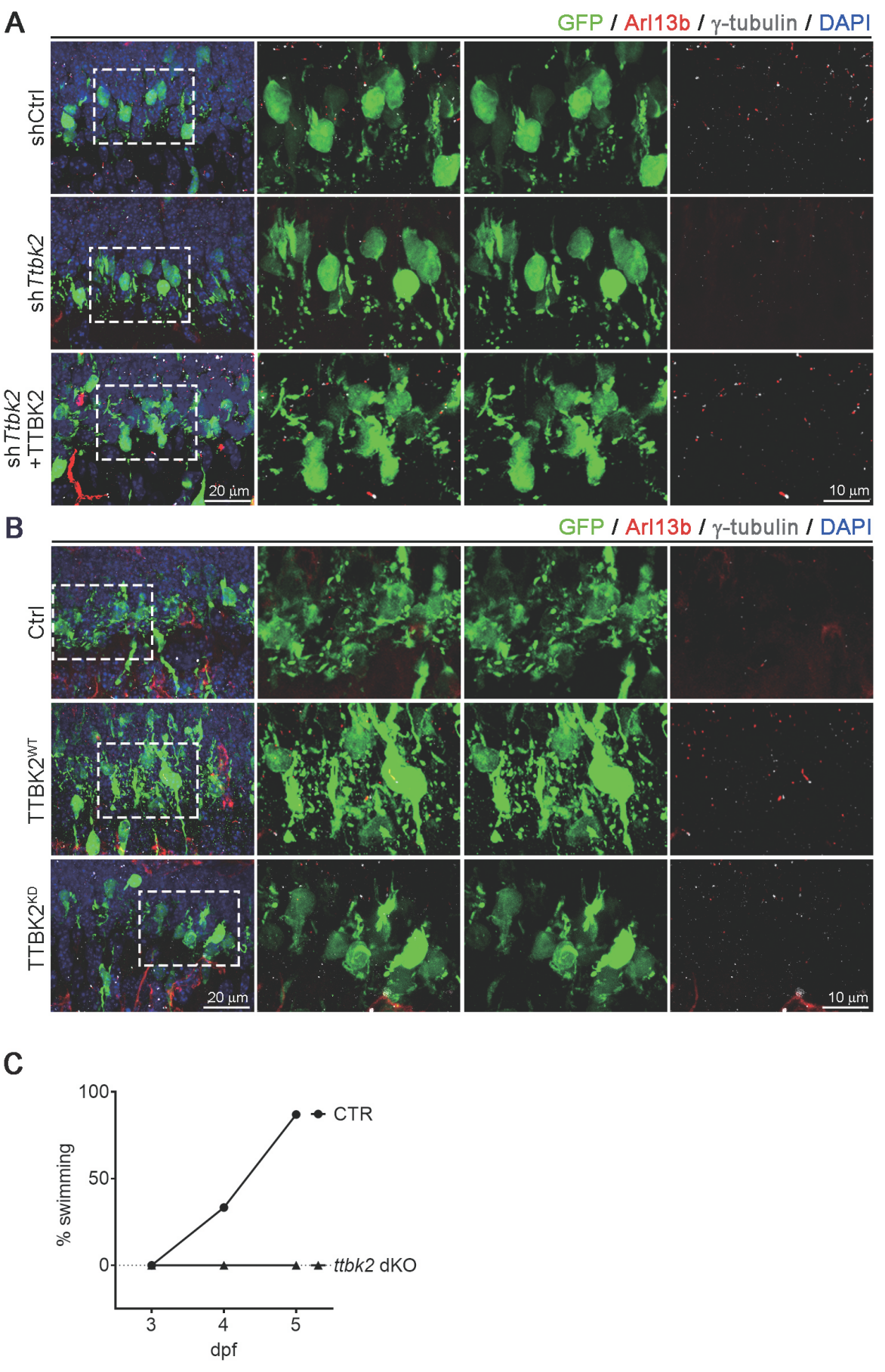


**Supplemental Fig. 2 Ttbk2 regulates GNP proliferation. A** GNPs in EGL of P6 mouse were electroporated with plasmids of shCtrl, sh*Ttbk2* or sh*Ttbk2* in combination with human TTBK2 for 2 days. Immunostaining was performed to label electroporated cells (GFP; green), cilia (Arl13b; red) and centrosome (γ-tubulin; white). Nuclei were stained by DAPI (blue). Scale bar are as indicated. Regions within the marked boxes were magnified and shown in right. **B** GNPs in EGL of P6 mouse were electroporated with plasmids of control, TTBK2^WT^ or TTBK2^KD^ for 2 days. Immunostaining was performed to label electroporated cells (GFP; green), cilia (Arl13b; red) and centrosome (γ-tubulin; white). Nuclei were stained by DAPI. Scale bar are as indicated. **C** The ratio of the fish capable swimming during early developing stages was quantified. The CTR fish are siblings of the dKO fish resulting from the mating of *ttbk2a^+/-^*; *ttbk2b^-/-^* parent fish. The percentage of these CTR sibling fish that start swimming is represented by dots (n=46), while the *ttbk2* dKO fish are marked by triangle (n=19).


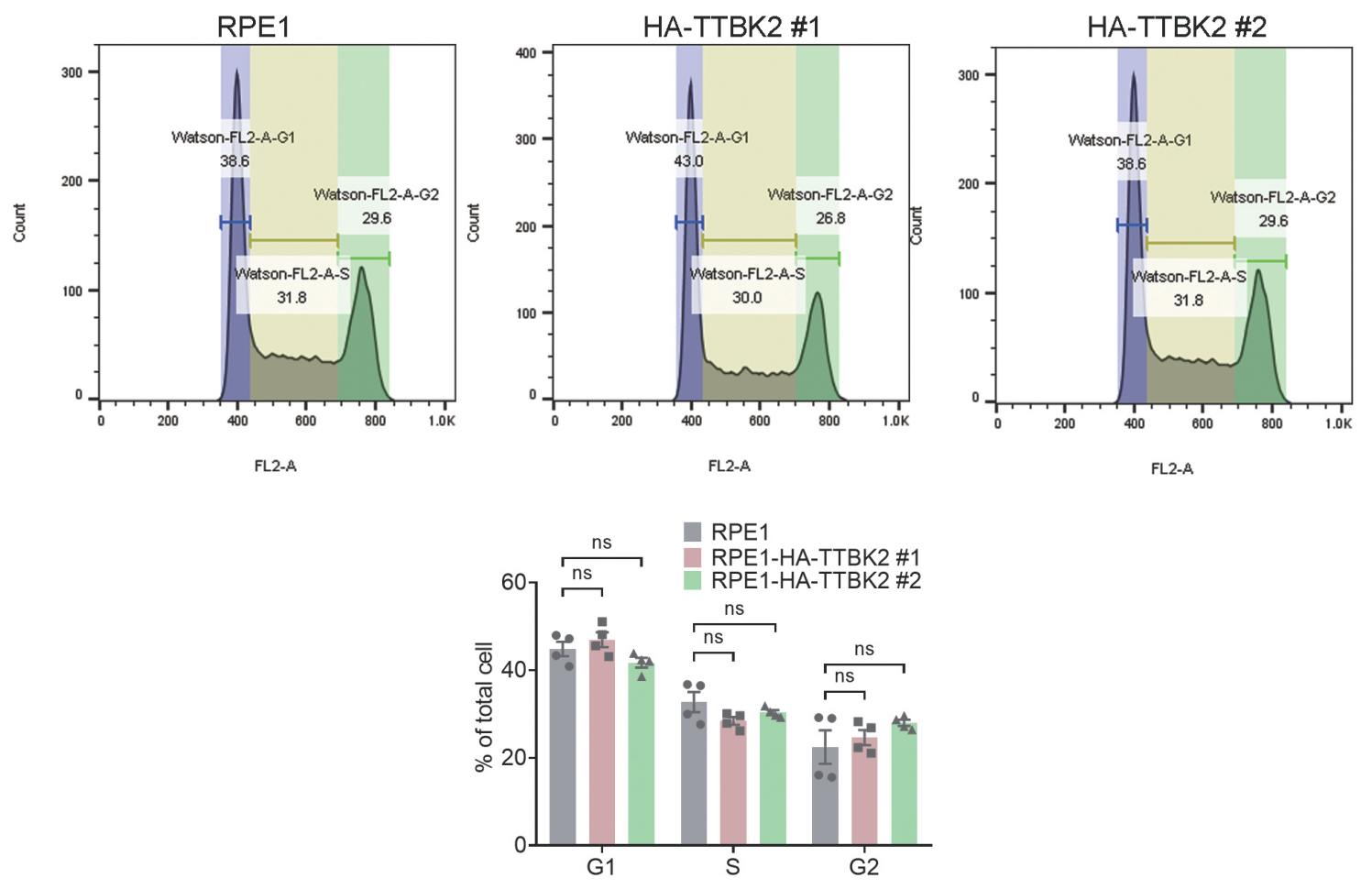


**Supplemental Fig. 3 TTBK2 overexpression does not affect cell-cycle progression.** Cell cycle distribution in the control and two TTBK2-overexpressing RPE1 cell lines were shown in terms of mean ± SEM of data from n=4 independent experiments. ns, not significant by Student’s t test.


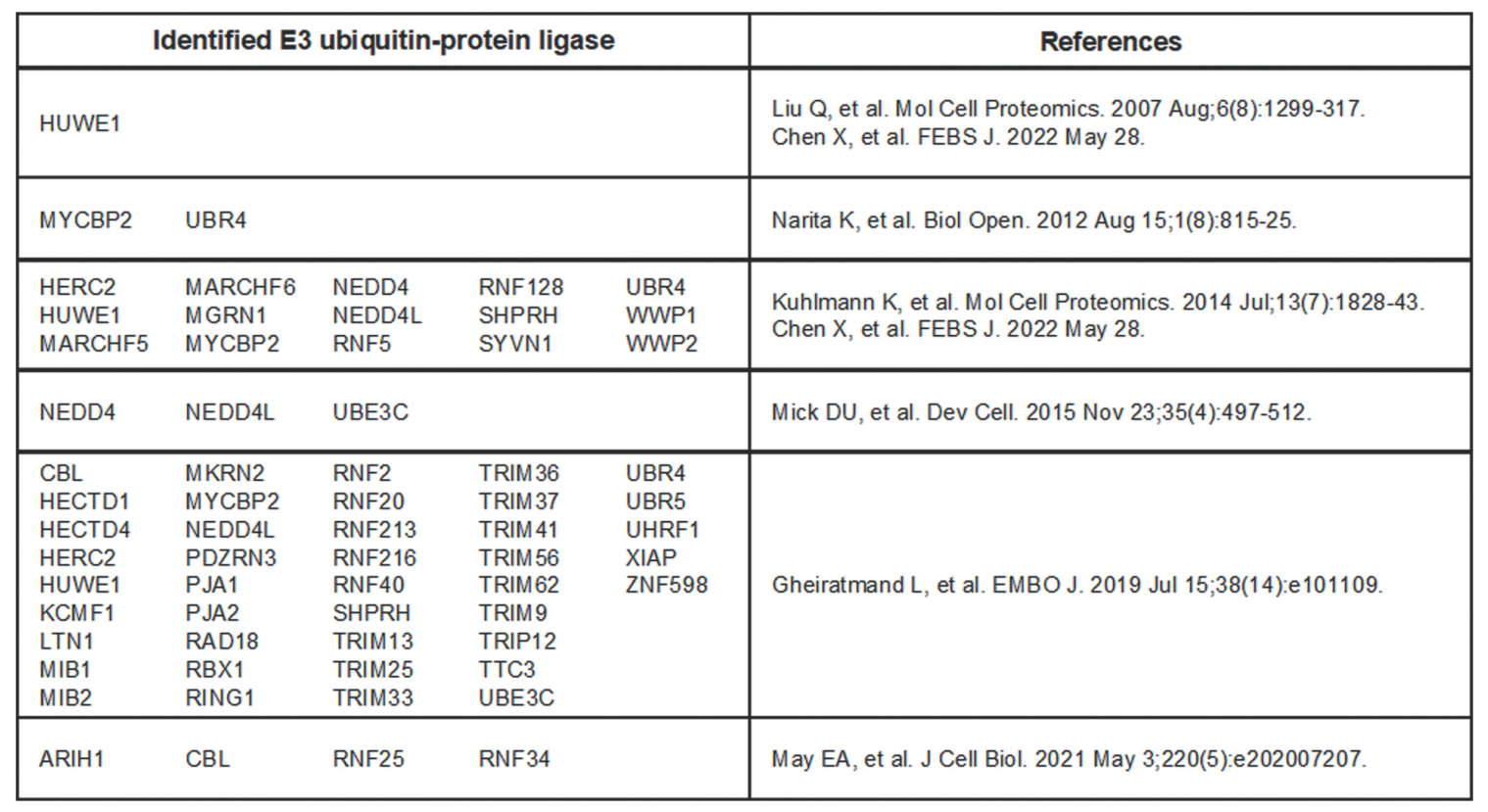


**Supplemental Fig. 4 Summary of candidate E3 ligases in the centrosome and cilia proteome.** The summary table showing E3 ligases are identified in the centrosome and cilia proteomes.


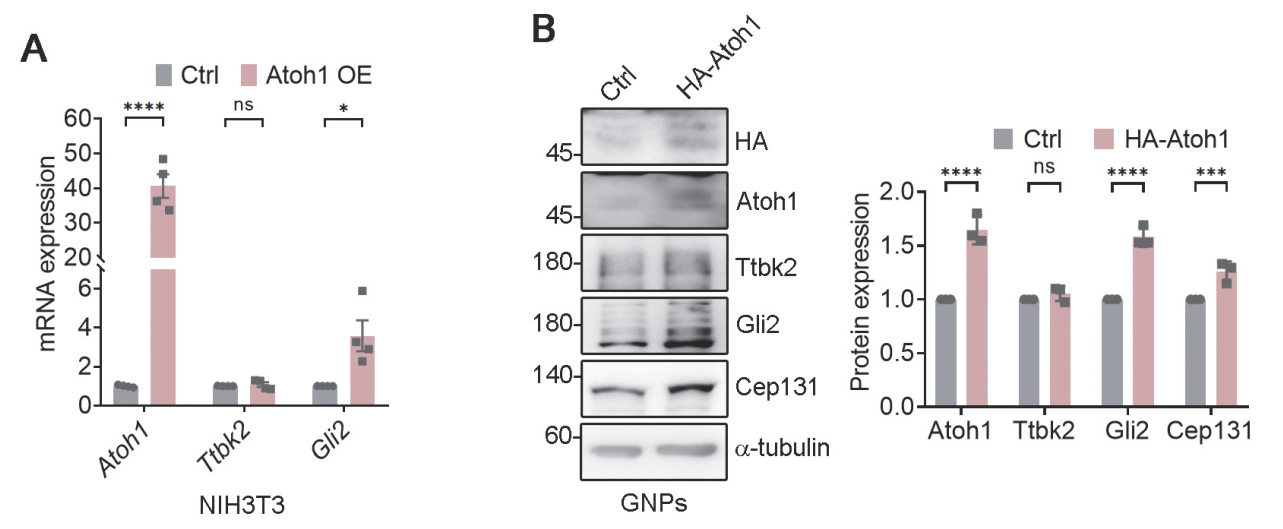


**Supplemental Fig. 5 Ttbk2 degradation promoted by Huwe1 in GNPs is Atoh1 independent. A** qPCR was performed to examine *Atoh1*, *Ttbk2* and *Gli2* levels in Atoh1 OE cells. Data were normalized to an internal control (18S). **B** HA-tagged-Atoh1 was ectopically expressed in purified GNPs. The levels of Atoh1, Ttbk2, Gli2, and Cep131 were examined by immunoblots and quantified. Error bars represent the mean ± SD from n=3 independent experiments. ns, not significant, ****p<0.0001, ***p<0.001 by Student’s t test.


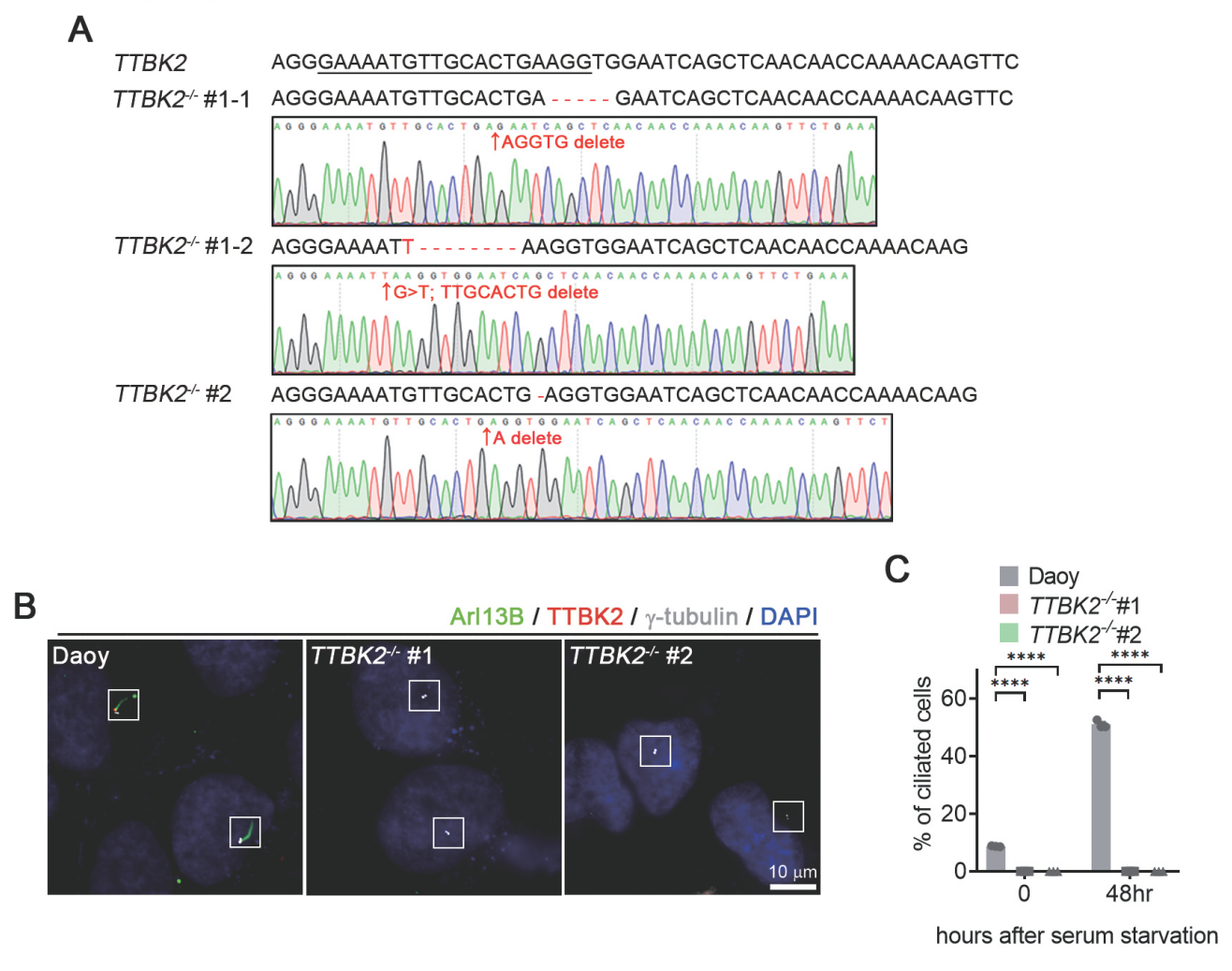


**Supplemental Fig. 6 Generation of *TTBK2* knockout Daoy cells. A** Sequence analyses of TTBK2 alleles in two *TTBK2^-/-^* Daoy cell lines were shown. The TTBK2 gRNA sequence is underlined and the lesion is shown in red. **B** Immunostaining was performed in the control and two *TTBK2^-/-^* Daoy cells using antibodies as indicated. Nuclei were stained by DAPI. Regions within the marked boxes were magnified and shown in Fig. 7G. Scale bar, 10 μm. **C** Cells were serum starved to induce cilia formation. The ciliated frequency was determined by Arl13b staining. Data were collected from n=3 independent experiments. Error bars represent the mean ± SEM. ****p < 0.0001 by Student’s t test.


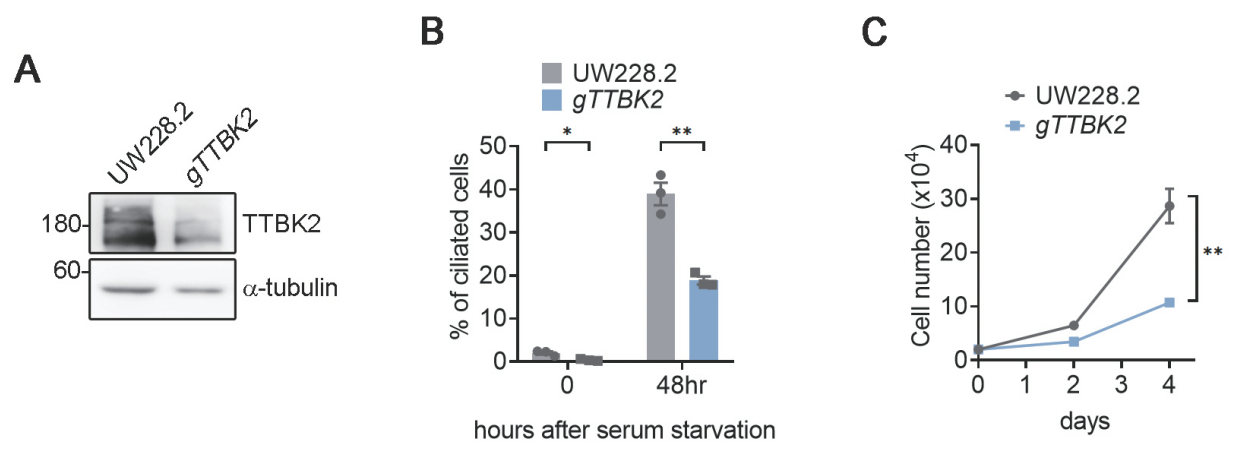


**Supplemental Fig. 7 TTBK2 depletion inhibits cell proliferation in UW228.2 cells. A** UW228.2 cells were transfected with the control or TTBK2 gRNAs. Immunoblots were performed with anti-TTBK2 and anti-α-tubulin antibodies. **B** Cells were serum starved to induce cilia formation. The ciliated frequency was determined by Arl13b staining. Data were collected from n=3 independent experiments. Error bars represent the mean ± SEM. *p < 0.05, **p < 0.01 by Student’s t test. **C** Cell proliferation assay was performed from n=3 independent experiments. Error bars represent the mean ± SEM. ****p<0.0001 by Two-way ANOVA.

**SUPPLEMENTARY RRFERENCES**

1. Zhao H, Ayrault O, Zindy F, Kim JH, Roussel MF. Post-transcriptional down-regulation of Atoh1/Math1 by bone morphogenic proteins suppresses medulloblastoma development. *Genes Dev* 2008, **22**(6)**:** 722-727.

2. Lee HY, Greene LA, Mason CA, Manzini MC. Isolation and culture of post-natal mouse cerebellar granule neuron progenitor cells and neurons. *J Vis Exp* 2009(23).

3. Chang CH, Zanini M, Shirvani H, Cheng JS, Yu H, Feng CH*, et al.* Atoh1 Controls Primary Cilia Formation to Allow for SHH-Triggered Granule Neuron Progenitor Proliferation. *Dev Cell* 2019, **48**(2)**:** 184-199.e185.

4. Hunkeler M, Jin CY, Ma MW, Monda JK, Overwijn D, Bennett EJ*, et al.* Solenoid architecture of HUWE1 contributes to ligase activity and substrate recognition. *Mol Cell* 2021, **81**(17)**:** 3468-3480.e3467.

5. Kani S, Bae YK, Shimizu T, Tanabe K, Satou C, Parsons MJ*, et al.* Proneural gene-linked neurogenesis in zebrafish cerebellum. *Dev Biol* 2010, **343**(1-2)**:** 1-17.

6. Lo CH, Lin IH, Yang TT, Huang YC, Tanos BE, Chou PC*, et al.* Phosphorylation of CEP83 by TTBK2 is necessary for cilia initiation. *J Cell Biol* 2019, **218**(10)**:** 3489-3505.

7. Ran FA, Hsu PD, Wright J, Agarwala V, Scott DA, Zhang F. Genome engineering using the CRISPR-Cas9 system. *Nat Protoc* 2013, **8**(11)**:** 2281-2308.

8. Thisse C, Thisse B. High-resolution in situ hybridization to whole-mount zebrafish embryos. *Nat Protoc* 2008, **3**(1)**:** 59-69.
