# Supplementary material for: Regulation of primary cilia disassembly through HUWE1-mediated TTBK2 degradation plays a crucial role in cerebellar development and medulloblastoma growth
Source: Cell Death Differ. 2024 Jun 15;31(10):1349–61. doi: 10.1038/s41418-024-01325-2 (PMC11445238; doi:10.1038/s41418-024-01325-2)

Supplementary original blots

Fig. 1A

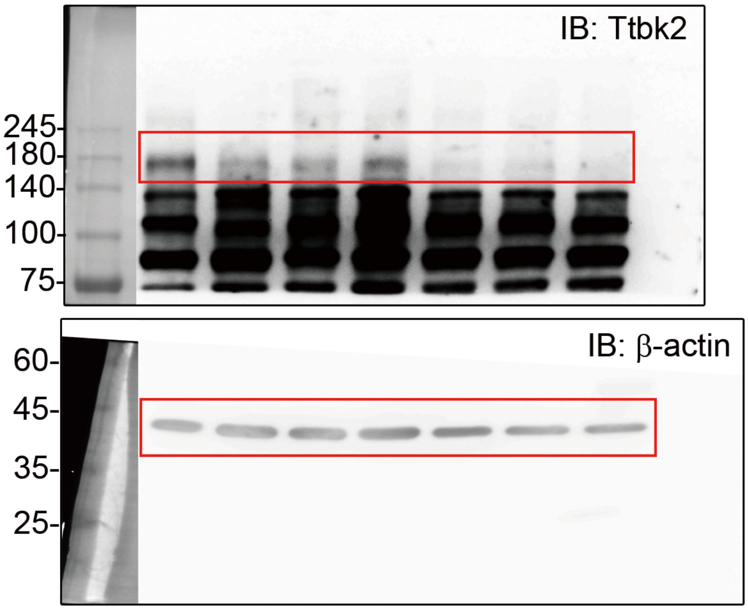

Fig. 1B

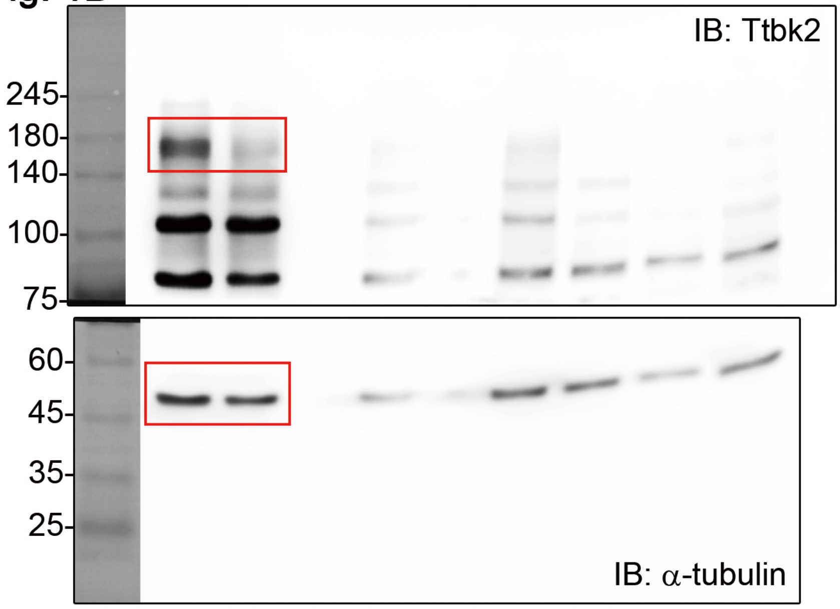

Fig. 1E

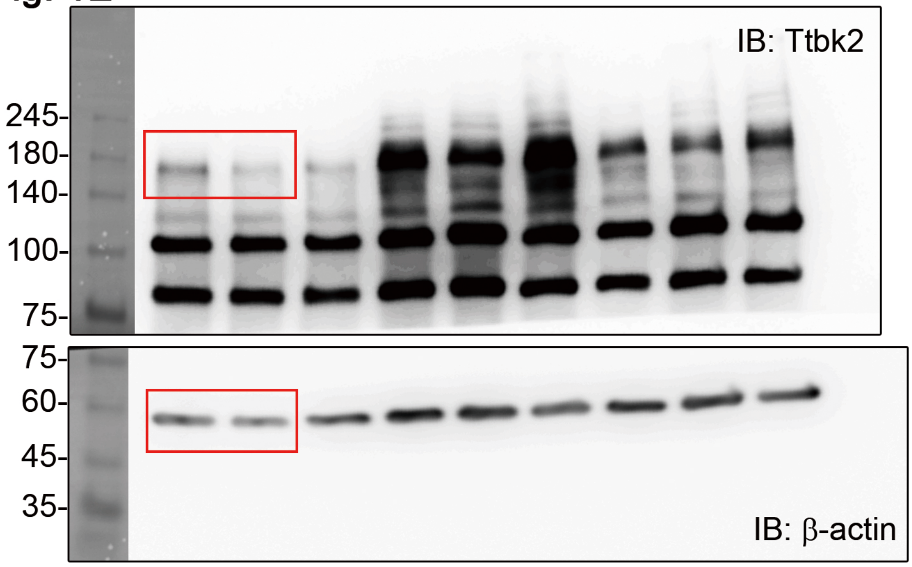

Fig. 3A

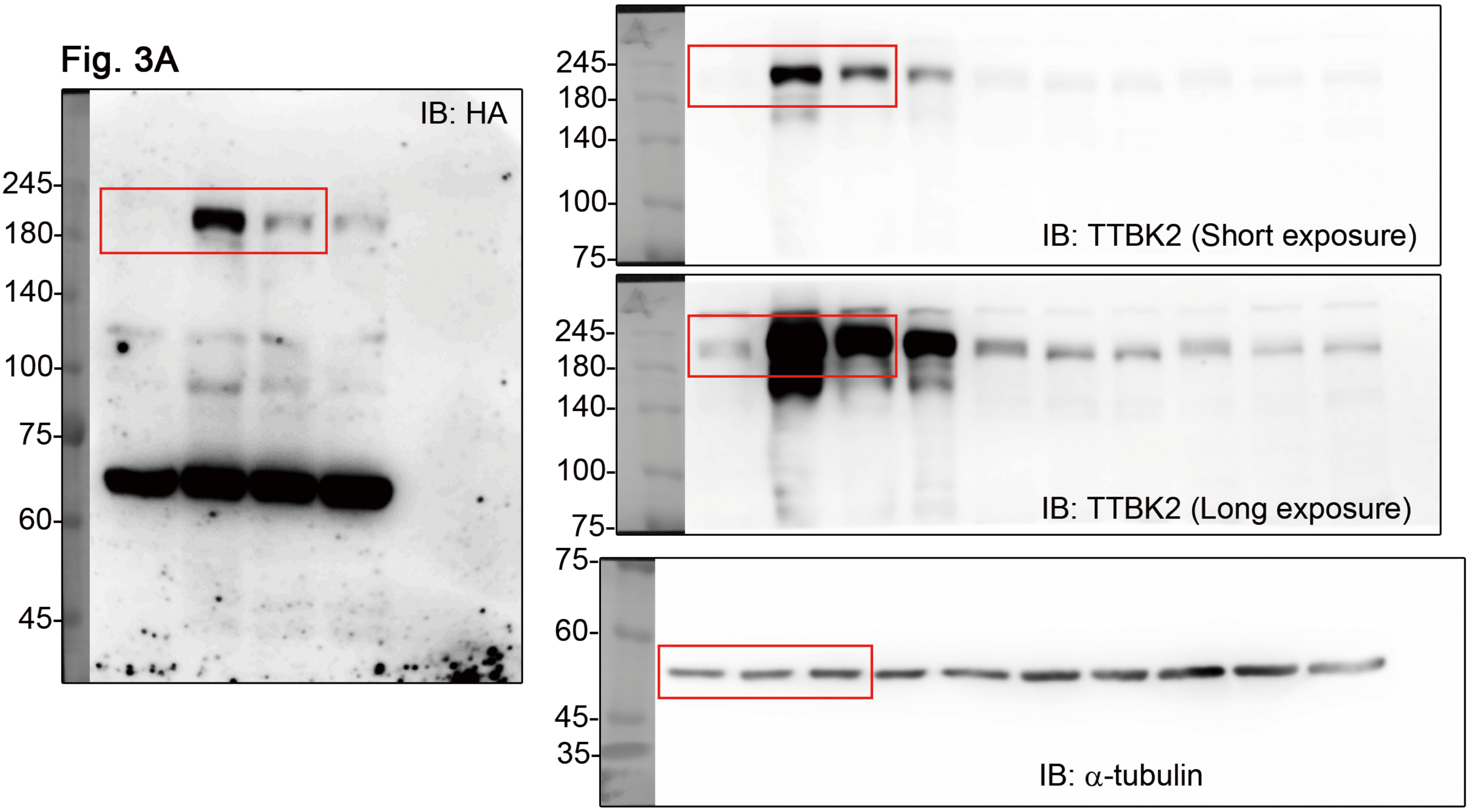
IB: HA
245-
180-
140-
100-
75-
60-
45-
245-
180-
140-
100-
75-
IB: TTBK2 (Short exposure)
245-
180-
140-
100-
75-
IB: TTBK2 (Long exposure)
75-
60-
45-
35-
IB: α-tubulin

Fig. 4B

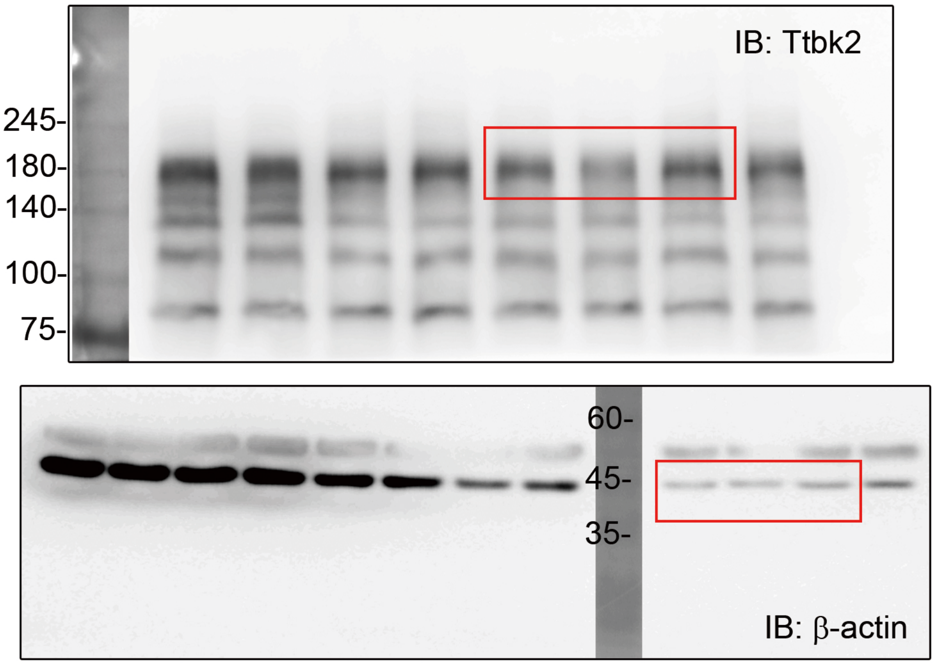
IB: Ttbk2
245-
180-
140-
100-
75-
60-
45-
35-
IB: β-actin

Fig. 4E

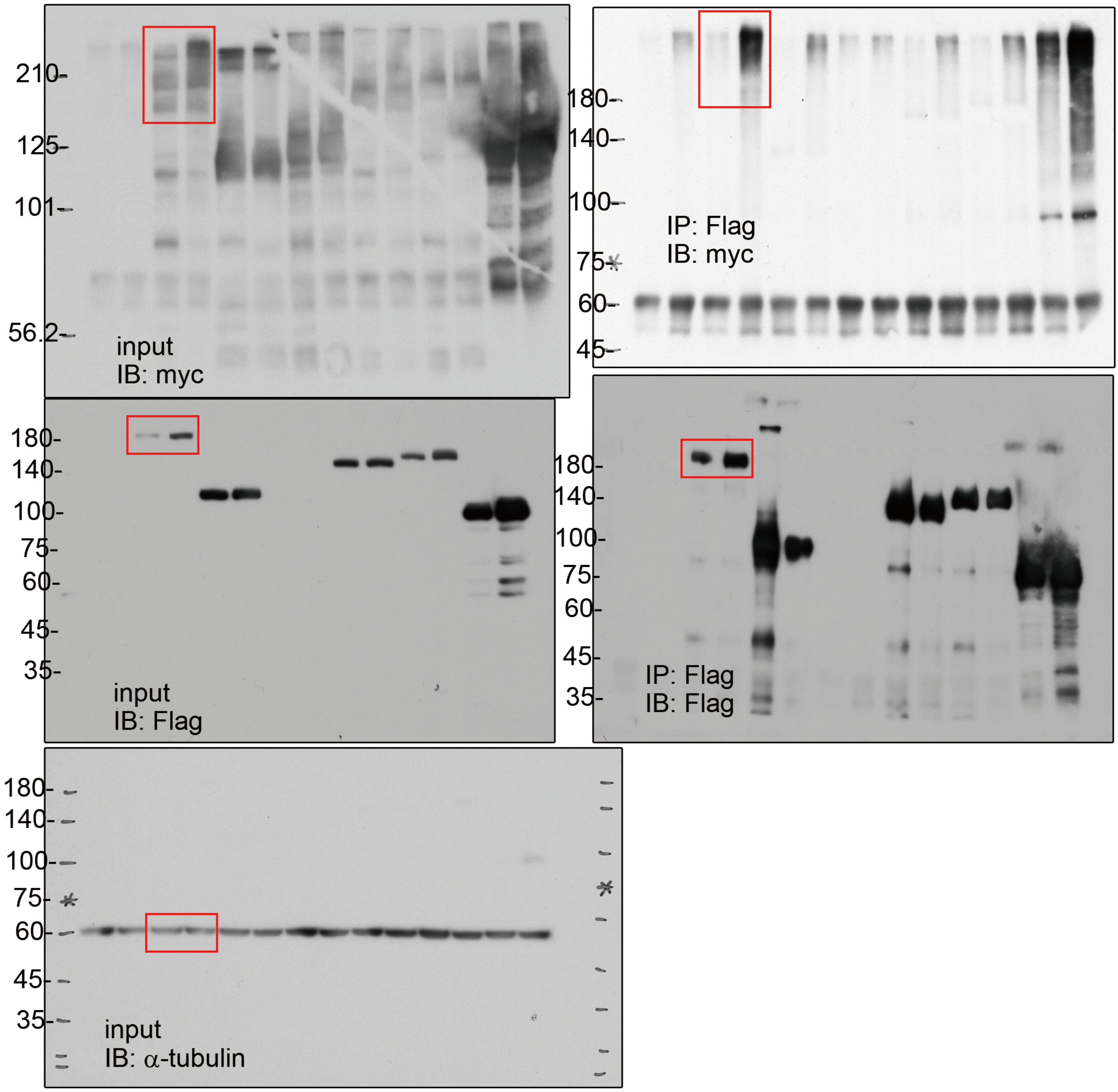
210-
125-
101-
56.2-
input
IB: myc
180-
140-
100-
75-
60-
45-
IP: Flag
IB: myc
180-
140-
100-
75-
60-
45-
35-
input
IB: Flag
180-
140-
100-
75-
60-
45-
35-
IP: Flag
IB: Flag
180-
140-
100-
75-
60-
45-
35-
input
IB: α-tubulin

Fig. 4F

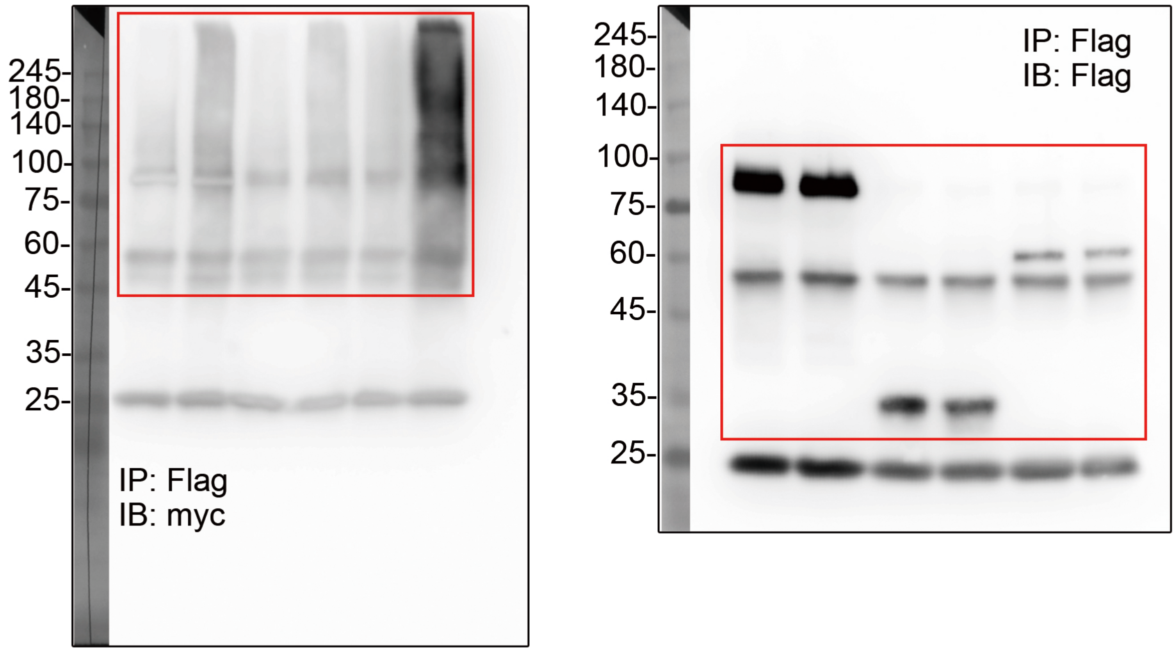
245-
180-
140-
100-
75-
60-
45-
35-
25-
IP: Flag
IB: myc
245-
180-
140-
100-
75-
60-
45-
35-
25-
IP: Flag
IB: Flag

Fig. 5B

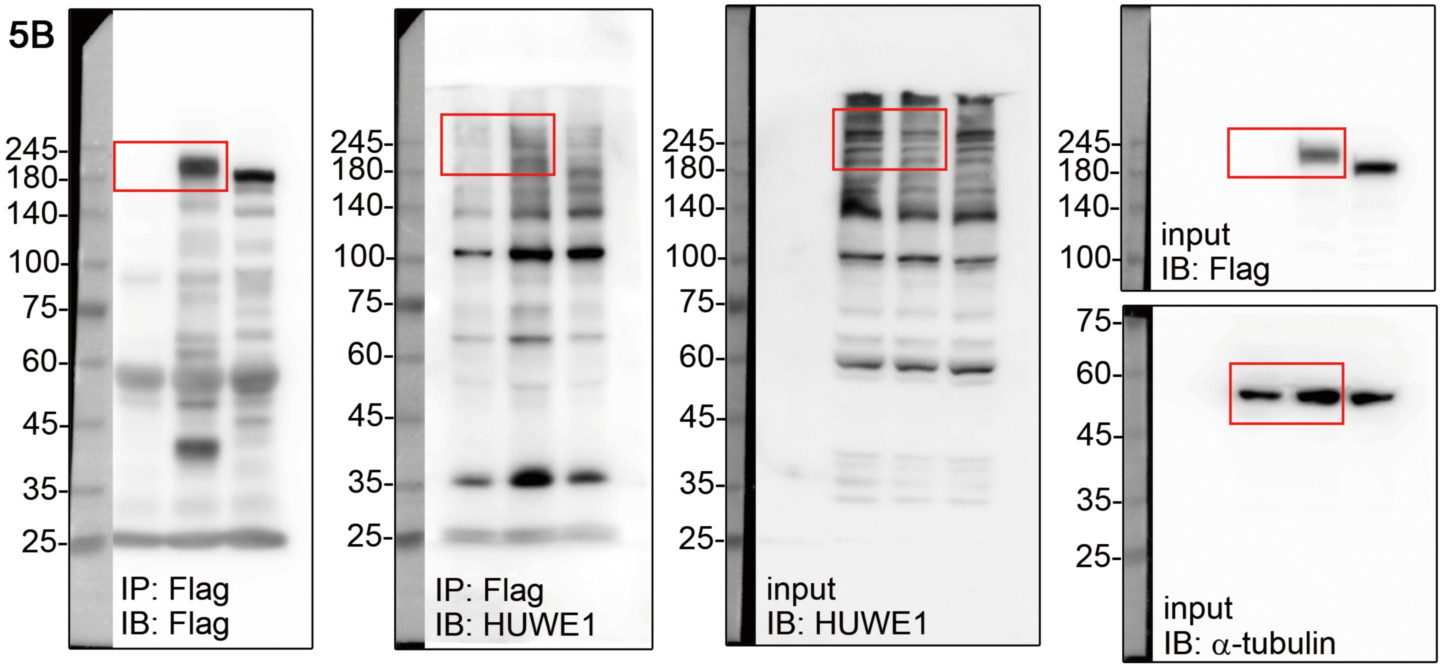

Fig. 5G

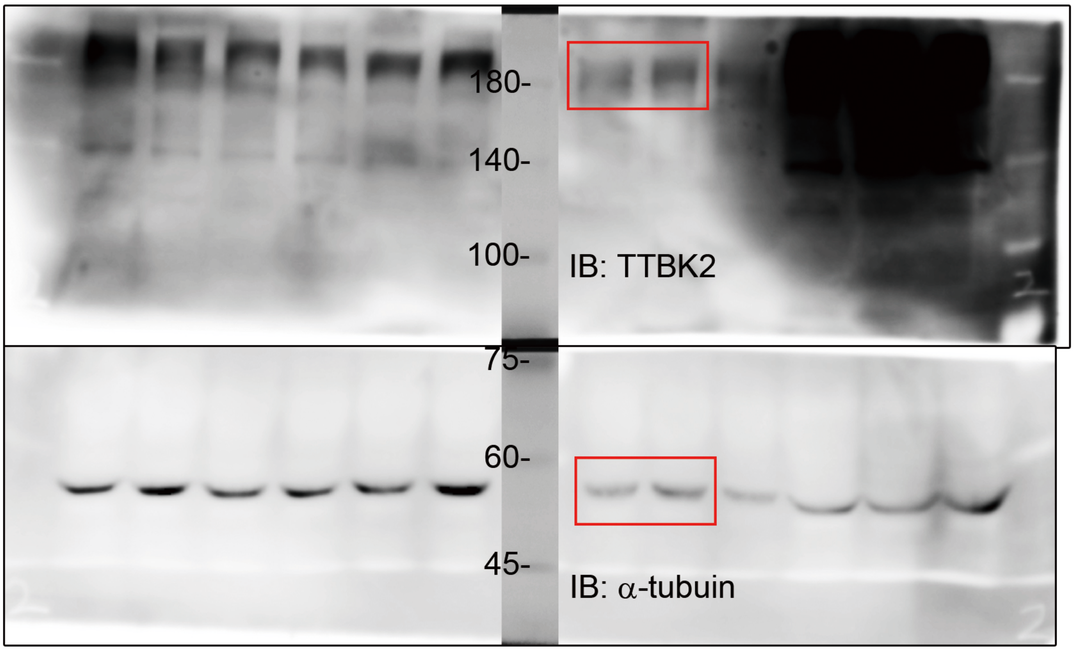

Fig. 5J

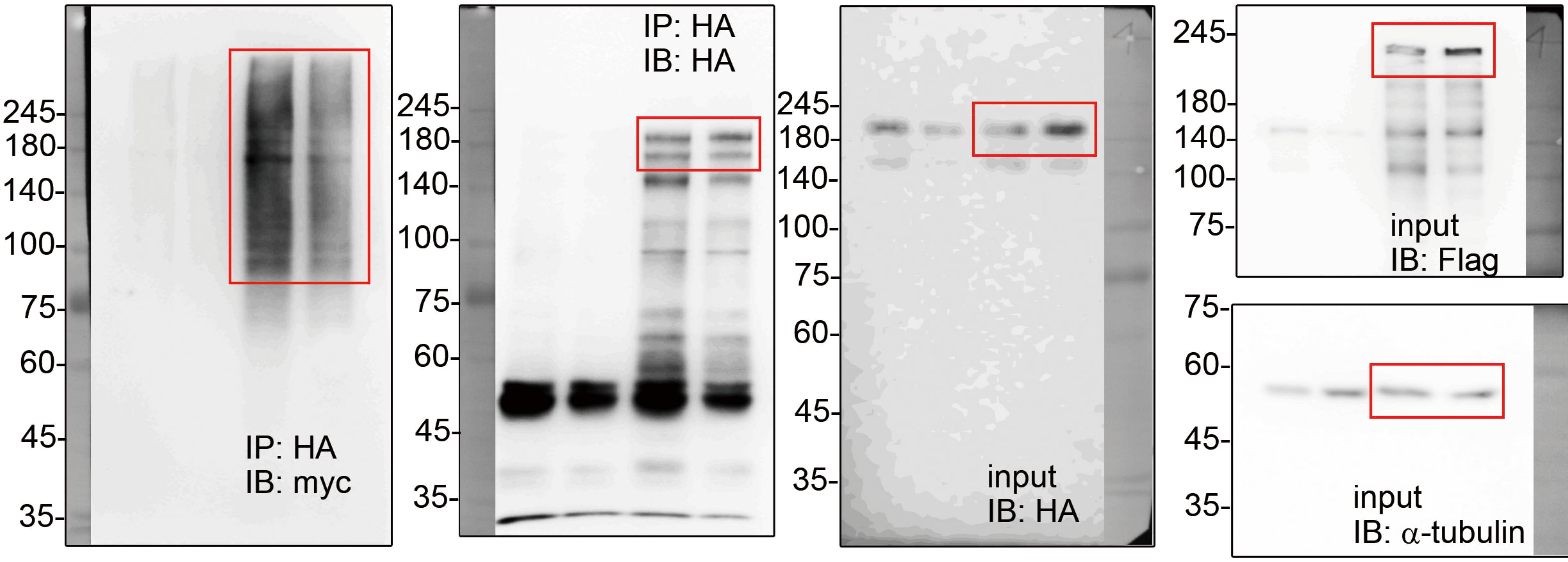

Fig. 6A

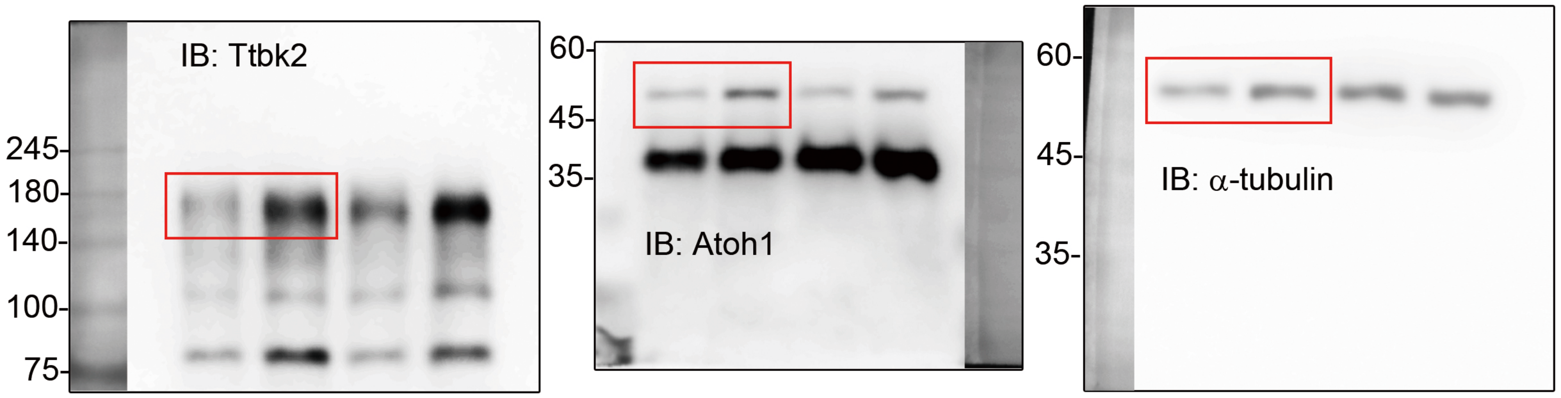

Fig. 6B

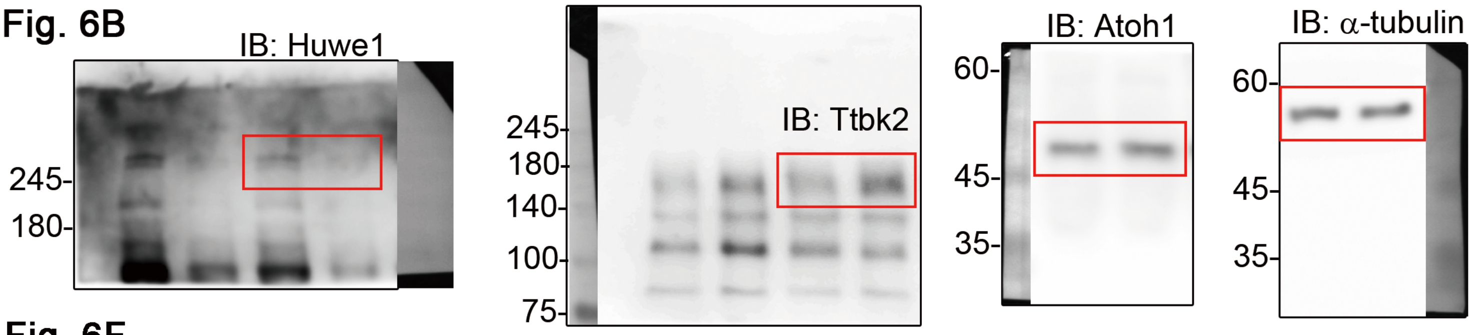

Fig. 6F

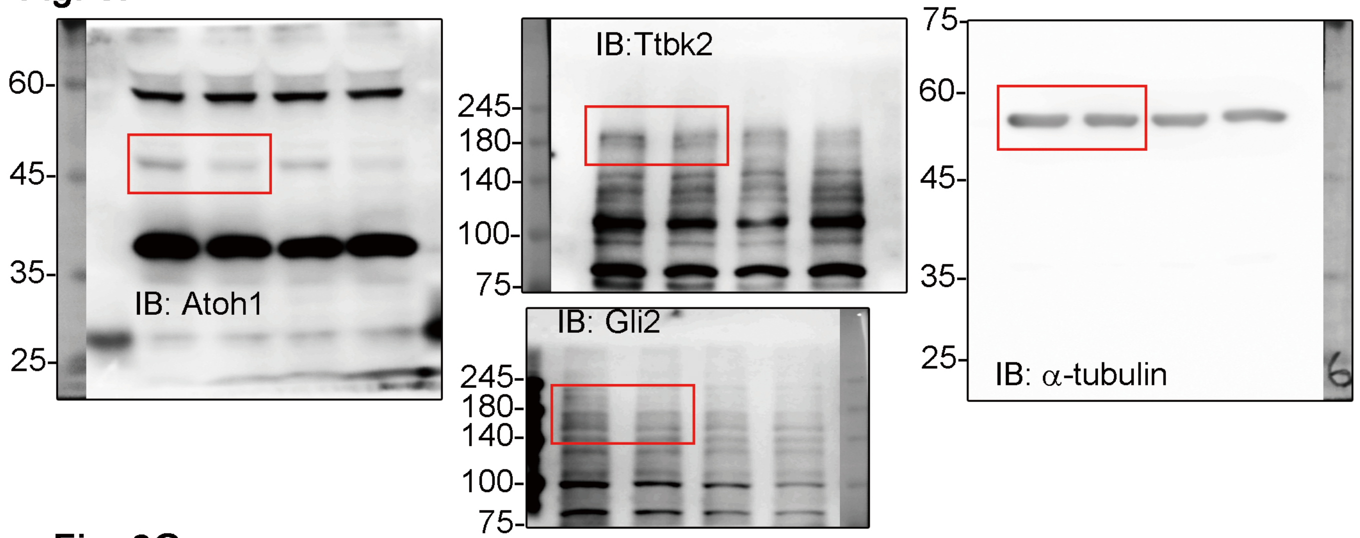

Fig. 6G

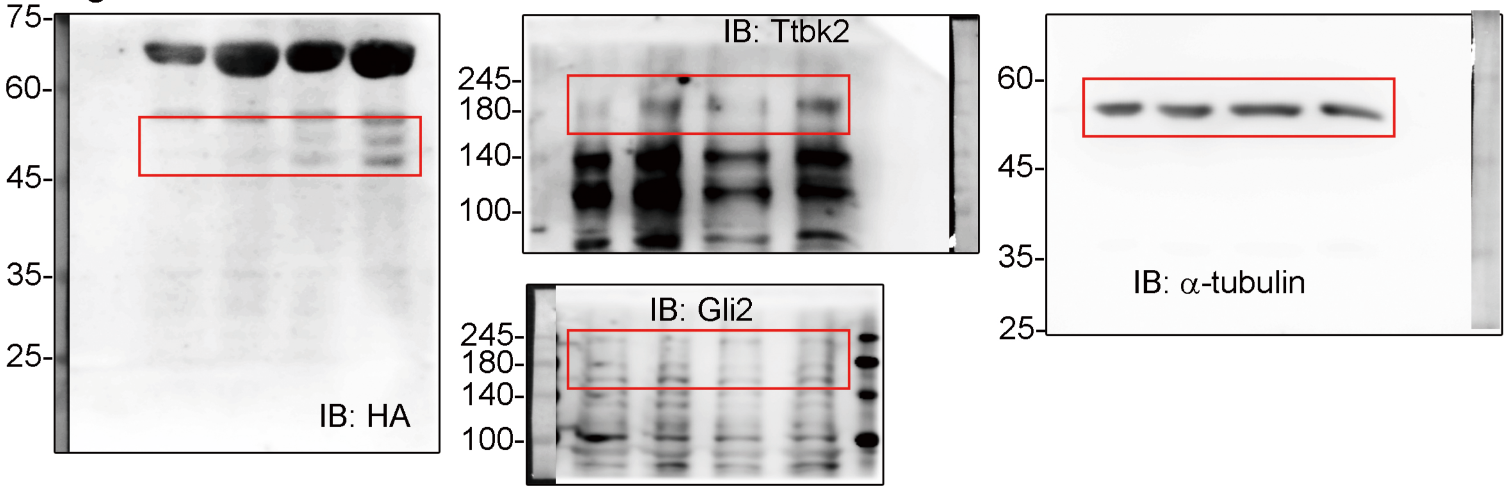

Fig. 7F

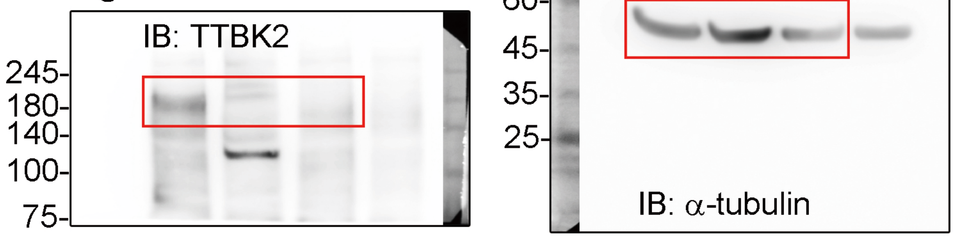

Fig. S5B

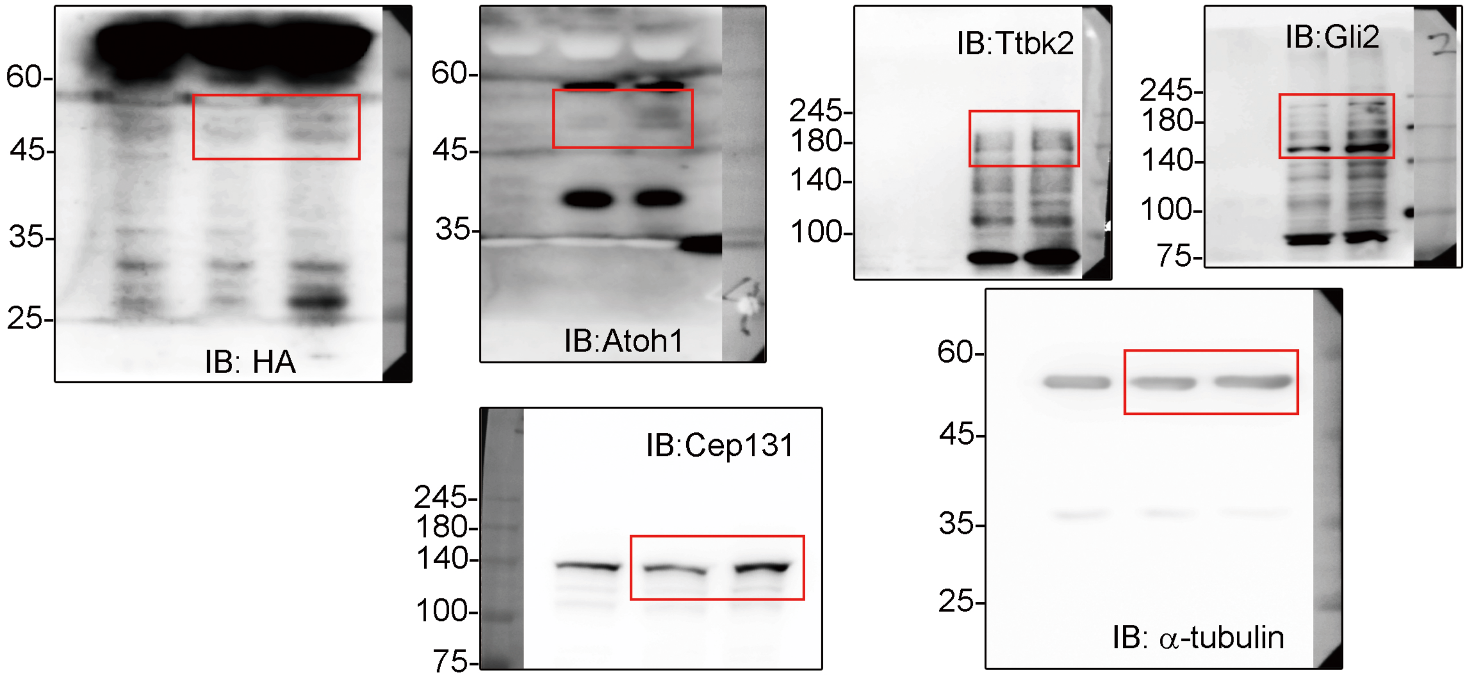

Fig. S7A

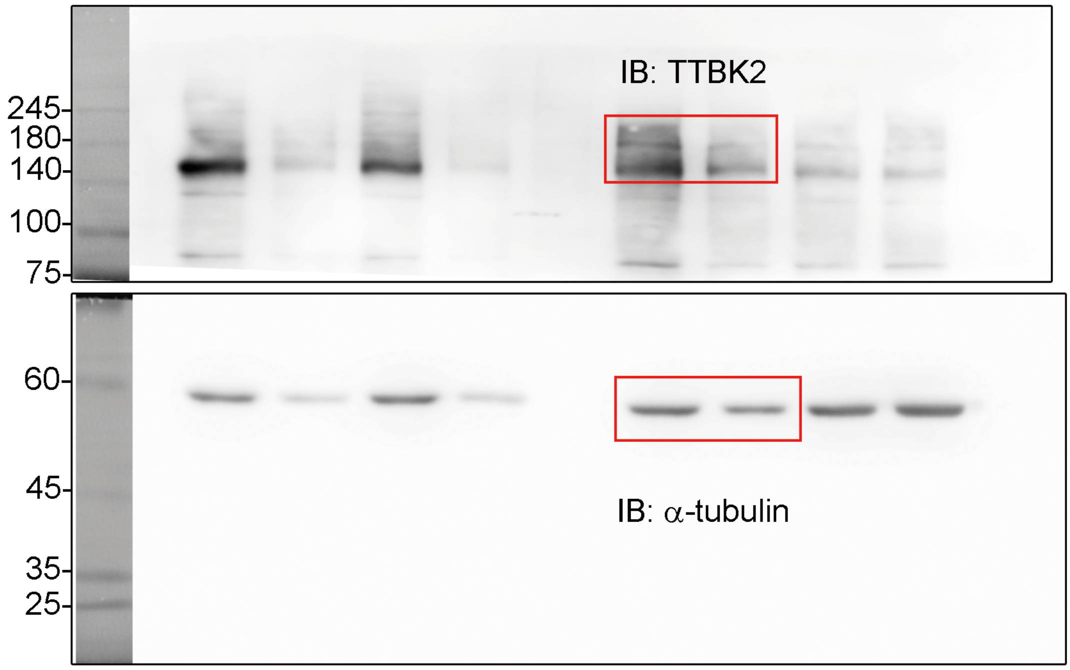

Supplement: Supplementary file 2 — Uncropped original blots [file 41418_2024_1325_MOESM2_ESM.pdf]
